# Scoping review of health promotion and disease prevention interventions addressed to elderly people

**DOI:** 10.1186/s12913-016-1521-4

**Published:** 2016-09-05

**Authors:** Mariusz Duplaga, Marcin Grysztar, Marcin Rodzinka, Agnieszka Kopec

**Affiliations:** Department of Health Promotion, Institute of Public Health, Faculty of Health Sciences, Jagiellonian University Medical College, Grzegorzecka Str. 20, 31-531 Krakow, Poland

**Keywords:** Elderly, Older adults, Health promotion, Primary disease prevention, Screening, Social support, Scoping review, Systematic review

## Abstract

**Background:**

The ageing of modern societies remains one of the greatest challenges for health and social systems. To respond to this challenge, we need effective strategies assuring healthy active life for elderly people. Health promotion and related activities are perceived as a key intervention, which can improve wellbeing in later life. The main aim of this study is the identification and classification of such interventions addressed to older adults and elderly. Therefore, the strategy based on the scoping review as a feasible tool for exploring this domain, summarizing research findings and identifying gaps of evidence, was applied.

**Methods:**

The scoping review relies on the analysis of previous reviews of interventions aimed at older adults (55–64 years old) and elderly persons (65 years and above) assessed for their effectiveness in the framework of a systematic review and/or meta-analysis. The search strategy was based on the identification of interventions reported as health promotion, primary disease prevention, screening or social support. In the analysis, the reviews published from January 2000 to April 2015 were included.

**Results:**

The search strategy yielded 334 systematic reviews and/or meta-analyses addressed to target groups of interest, 182 of them assessed interventions belonging to health promotion, 219 to primary prevention, 34 to screening and 35 to social support. The studies focused on elderly (65 years and above) made up 40.4 % of all retrieved reviews and those addressing population of 55 years and above accounted for 24.0 %.

**Conclusions:**

Interventions focused on health maintenance and improvement in elderly and older adults represent frequently combined health promotion and disease prevention actions. Many interventions of this type are not addressed exclusively to elderly populations and/or older adults but are designed for the general population. The most common types of interventions addressed to elderly and older adults in the area of health promotion include health education, behavior modification and health communication.

**Electronic supplementary material:**

The online version of this article (doi:10.1186/s12913-016-1521-4) contains supplementary material, which is available to authorized users.

## Background

Population ageing is perceived as one of the greatest challenges for modern societies both in terms of economic burden and social demands. In 2010, people aged 65 years and over made up 15 % of the overall population in Europe. Estimations indicate that in 2050 this figure will reach at least 25 % [1]. Maintaining health among older groups remains a demanding task. It is obvious that morbidity increases with age and multi-morbidity is more common in elderly populations [2].

As a response to this challenge, many policies and strategies on international, national or other levels have been formulated. On a general level, they are aimed at reaching goals related to affirmative concepts of ageing formulated as ‘active ageing’ [3], ‘healthy ageing’ [4], ‘productive ageing’ [5] or ‘positive ageing’ [6, 7]. According to the Policy Framework issued by the World Health Organization in 2002, the development of appropriate policies and programs that enhance the health, participation and security of older citizens is essential for meeting this challenge [3].

Strategies, which broadly fall into the domain of health promotion and disease prevention, bring a promise of a healthier and more productive life in advanced age. Health promotion is a relatively recent approach to improving the health of societies and individuals. To some extent, it has been developed as a response to the dissatisfaction with ongoing efforts in health care during the 1970s. During the First International Conference of Health Promotion held in Ottawa, Canada, in 1985, health promotion was defined as “the process of enabling people to increase control over, and to improve, their health” [8]. It was included in the Ottawa Charter perceived as one of the key documents establishing the basis for health promotion as a domain. The Charter also specifies the five main action types for health promotion. They encompass building healthy public policies, creating supportive environments, strengthening community actions, developing personal skills, and reorienting health services.

In turn, disease prevention is usually perceived as a complementary term to health promotion, although its definitions focus on the context of avoiding diseases or their consequences, and not on the concept of health. According to the Health Promotion Glossary, disease prevention encompasses “measures not only to prevent the occurrence of disease, such as risk factor reduction, but also to arrest its progress and reduce its consequences once established” [9]. Primary disease prevention is aimed at precluding the onset of disease. Secondary prevention should lead to controlling the disease before it manifests clinically. Screening is an example of such measure. In patients with a developed disease, tertiary prevention may be undertaken in order to decrease its impact on the patient’s life [10].

Although health promotion and disease prevention are treated as separate concepts, the difference is less visible when we consider practical applications. To some extent, health promotion may be perceived as being aligned with positive definitions of health extending beyond the absence of disease. Health promotion may be seen as a broader concept supporting the achievement of wellbeing and happiness. In turn, disease prevention aims to avoid or eliminate diseases. Health promotion does not need to involve disease prevention, but disease prevention cannot be achieved without health promotion [11]. A close relationship between health promotion and disease prevention may result in some difficulties in the classification of interventions focused on the maintenance and improvement of health.

The aim of this paper is the identification and classification of health promotion and related types of interventions addressing general health issues as well as those specific to ageing among older adults and elderly people. For this purpose, the framework of a scoping review was applied based on the analysis of systematic reviews and/or meta-analyses focused on the assessment of effectiveness of relevant interventions.

To authors’ knowledge, such review of secondary evidence on interventions promoting or adding to health of elderly persons was not done before. It is also anticipated that accumulated secondary evidence in this domain may be used for formulating policy recommendations on the effectiveness of interventions related to the maintenance and improvement of health in these populations. The broad view of the domain should also reveal potential gaps in secondary evidence and navigate researchers to these areas, which should be addressed in future systematic reviews.

The focus of the scoping review was on health promotion addressed to elderly or older adults; however, a rigid extraction of health promotion interventions from other related actions, especially disease prevention, could artificially limit the scope of efficient types of interventions focused on the maintenance of health and avoiding health risks in elderly people. To avoid this limitation and taking into consideration the frequent combined use of the terms of health promotion and disease prevention in effectiveness reports, a broad strategy of retrieving secondary evidence has been established. It has been deliberately extended to the three additional concepts including primary disease prevention, screening and social support to obtain a better view of actions aimed at improving health in elderly people.

The research question established for the scoping review was formulated as follows: “What types of interventions promoting the health of the elderly population have been assessed for their effectiveness in systematic reviews and meta-analyses?” The review focuses on interventions addressed at healthy older adults and elderly people, or on interventions focusing on general health issues of these groups even if they suffer from specific disorders.

Definitions of health promotion remain general or tend to favor selected types of interventions or outcomes. Although health is usually stated or regarded as the default aim, the instrumental objectives (following elements of the health promotion definition proposed by Rootman in 2001 [12]), processes or actions are not stated systematically. As the definition proposed in the Ottawa Charter [8], repeated in the WHO glossary [9], is the most widely recognized, the review reported in this paper used it as a guiding statement. Nevertheless, for further classification of possible interventions falling in the domain of health promotion, the taxonomy described by McKenzie et al. was used [13].

As a rule, the scoping review was focused on previous reviews of interventions aimed at general health issues or primary prevention of conditions not yet diagnosed in the target groups. However, it was also assumed that general areas of interventions could be relevant for individuals with diagnosed and treated medical conditions.

## Methods

### Study design

The study was based on the methodology of scoping review designed in order to identify and review the secondary evidence on the effectiveness of interventions addressing older adults and elderly people in the domain of health promotion and related areas. The research question for the scoping review was introduced earlier in the Background section. The scoping review is defined as “a process of mapping the existing literature or evidence base” [14]. According to Armstrong et al., it may be used to identify research gaps and summarize research findings, as well as to explore the extent of the literature in a particular domain, helping to identify appropriate parameters and defining a potential scope of a systematic review and the associated costs [15]. In contrast to the systematic review, the scoping review is generally characterized by broad research questions.

The design applied in this study anticipated the analysis of systematic reviews published between January 2000 and April 2015. It is assumed that the results of this review would be explored further with the aim of identifying effective health promotion and related interventions addressed to the elderly population and formulating recommendations on the policy level.

### Inclusion criteria

The scoping review described in this paper was based on the secondary analysis of available systematic reviews and/or meta-analyses. No other types of evidence were included. The main rationale for such approach was the attempt to obtain a view of interventions addressed to elderly people and older adults which underwent an assessment as to their effectiveness. The term “intervention” was applied in the meaning proposed by Rychetnik et al. as “an intervention comprises an action or program that aims to bring about identifiable outcomes” [16].

The systematic reviews and/or meta-analyses included in the scoping review met the following criteria: 1) the study assessed the effectiveness of health promotion or related interventions (primary prevention, screening, social support); definitions of these areas are included in the list contained in Additional file 1. The concept of effectiveness was used in line with the definition proposed by Wojtczak as “a measure of the extent to which a specific intervention, procedure, regimen, or service, when deployed in the field in routine circumstances, does what it is intended to do for a specified population. In the health field, it is a measure of output from those health services that contribute towards reducing the dimension of a problem or improving an unsatisfactory situation” [17], 2) the age of the target audience was at least 55 years old, or the target audience included subjects aged 55 years and above, 3) publication period was from January 2000 to April 2015, 4) published in English. Interventions related to therapy, diagnostics or rehabilitation required for specific diseases were excluded from the analysis. Systematic reviews whose key audiences were elderly individuals suffering from specific diseases were included in the scoping review providing that the interventions were aimed at general health issues and not specific symptoms or consequences of diseases diagnosed in these audiences.

### Search strategy

The search strategy was developed in order to identify systematic reviews and/or meta-analyses assessing the effectiveness of health promotion and related interventions addressed to elderly and older adults. The search strategy was based on the scheme derived from the classical PICO algorithm. The keywords included in the search are presented in Table 1. The search was performed in the following databases: MEDLINE, CINAHL, the Cochrane Library, EMBASE, INSPEC, PubPsych and ERIC.

**Table 1:** Keywords used in the search for secondary evidence

Population	Intervention/interest		Comparison	Outcome
Elderly	Health promotion and related areas		Systematic review	Effectiveness
Elderly Senior Senioral Elders Elder “Senior citizen” “Old age” “Old people” Seniors “Advanced age” Geriatric Aged Ageing Aging	“Health promotion” Prevention Intervention Interventions Campaign Campaigns “Health programme” “Health program” “Social support” “Social care” “Social intervention” Screening Preventive Prophylaxis Nutrition “Physical activity” Habits Addiction	“Health education” “Health literacy” “Health communication” “Health advocacy” “Community advocacy” “Social campaign” “Social campaigns” “Health coaching” “Environmental change strategies” “Healthy environment” “Community mobilization” “Behaviour modification” Prophylaxis Screening “Primary prevention” “Health screening” “Support groups” “Social network” “Social gathering” “Health changes”	“Systematic review” “Meta analysis” “Meta-analysis” Metaanalysis	Effectiveness Efficacy Efficiency Impact Evidence Outcomes

### Data extraction and assessment

Systematic reviews identified in the process were described according to criteria including year of publication, age and sex of targeted audiences, general areas of interventions, targeted areas of interventions, and in the case of interventions, classified as including health promotion actions, according to McKenzie et al. [13]. Four general areas of interventions were established to classify the papers retrieved in the search strategy described earlier. These areas encompassed health promotion, primary disease prevention, screening and social support.

The search strategy assumed the retrieval of interventions defined by authors in literature databases as disease prevention, although the selection of secondary evidence was guided by a rule that only primary prevention interventions were retrieved for the scoping review. Screening is usually classified as a specific type of intervention belonging to secondary prevention [10]. As it is aimed at finding disease (or risk factors) at an early stage in subjects who are not aware of their medical condition, it was included in the scoping review. Actions addressed to the community or undertaken in the community are of key importance for health promotion. It also seems that the support from social services and social care is particularly pertinent to the needs and situations of elderly people. Thus, interventions described as social support were selected as another category of a general area of interventions used for the classification of systematic reviews.

For the classification of the target area of interventions, a list of areas was developed first, including general health issues such as physical activity or nutrition, and areas specifically related to older age, e.g. frailty. This list was expanded with a few terms resulting from an initial analysis of papers retrieved.

The classification and description of secondary evidence was not made on the basis of its classification in literature databases or the keywords used for its selection, but it was carried out independently after the identification of feasible studies in the following process guided by existing definitions (applied definitions with sources are included in the Additional file 1). It means that even if the authors of a specific systematic review declared it as focusing on one of four main areas, e.g. health promotion, it could be re-classified by the authors of this scoping review according to definitions established for categories within classification dimensions.

Retrieved reviews were also classified according to the age of audience targeted by analyzed interventions. Four age categories were used: 1) 65 years of age or more – for interventions targeting exclusively elderly persons, 2) 55 years of age or more – for reviews analyzing interventions targeting both older adults and elderly persons, 3) general population including elderly persons – for reviews assessing interventions directed to general populations which could include elderly persons but without clear differentiation of results according to age categories, and finally 4) ‘other’ – for reviews which assessed interventions addressed to age groups addressed in other way but which included also elderly persons. The main rationale for how the age categories were structured, was related to an attempt of distinguishing interventions that were addressed specifically to elderly persons from these which were designed for broader age groups.

The classification process was conducted by two authors independently and divergent opinions were solved on the consensus basis. If a consensus was not reached, a third author was referred to for final decision.

The data collection tool used in this study was prepared as a form available to authors describing the retrieved studies on the www.esurv.org website. The results of the descriptions were exported to an Excel file. A descriptive analysis of the data was performed with Statistica v.10 PL (StatSoft, Tulsa, OK, USA) after importing the data from the Excel file.

We also provided the information about the quality of our review according to the PRISMA 2009 Checklist (see Additional file 2). Although this checklist was primarily designed for systematic review and/or meta-analysis, at least part of enlisted criteria may be applied to the scoping review.

## Results

### Search results

**Fig. 1:**
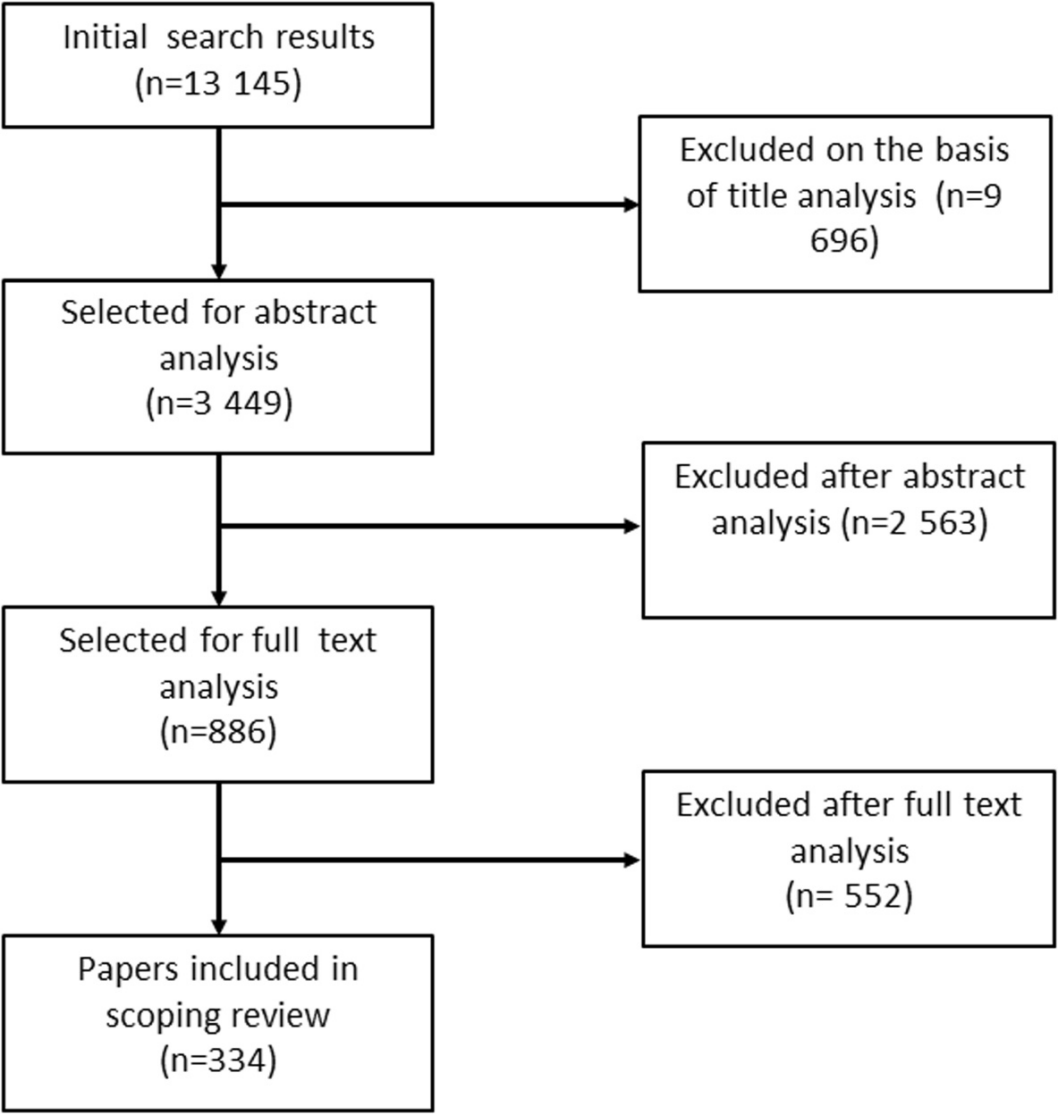
Flow diagram of the search strategy and study selection process

The search performed in the literature databases generated 13,145 papers, the verification based on the assessment of titles resulted in 3449 papers, and the analysis of abstracts limited the results to 886 papers selected for full-text assessment. The final stage, based on the analysis of full texts, resulted in the selection of 334 systematic reviews/meta-analyses for description and classification (Additional file 3). The flow diagram showing the whole search process is shown in Fig. 1.

### General area of intervention

From 334 retrieved systematic reviews, 182 were related to interventions classified as belonging to health promotion, 219 to primary disease prevention, 34 to screening, and 35 to social support. Systematic reviews exclusively related to primary disease prevention interventions were the most numerous category of studies, making up 33.5 % (*n* = 112) of all systematic reviews. Studies related to the analysis of the effectiveness of interventions combining health promotion and primary disease prevention actions were the second most numerous category (*n* = 79, 23.7 %), with those focused on health promotion interventions coming in third (*n* = 75, 22.5 %). Studies analyzing other exclusive categories of interventions were less numerous and made approximately 20 % in total. The numbers of systematic reviews according to the exclusive categories of interventions (individual or combined) are presented in Table 2.

**Table 2:** Number of systematic reviews retrieved according to exclusive categories of the general area of intervention

Category of intervention	Number of studies	%
primary prevention	112	33.5
health promotion & primary prevention	79	23.7
health promotion	75	22.5
screening	22	6.6
health promotion & primary prevention & social support	14	4.2
health promotion & social support	9	2.7
primary prevention & screening	7	2.1
social support	6	1.8
prevention & social support	5	1.5
health promotion & screening	3	0.9
health promotion & primary prevention & screening	1	0.3
all 4 general areas	1	0.3
screening & social support	0	0.0
health promotion & screening & social support	0	0.0
primary prevention & screening & social support	0	0.0

### Age categories and gender of targeted audiences

The age group of subjects targeted by the interventions assessed in the systematic reviews was another criterion used for the description of publications retrieved. The reviews focused on interventions targeting the elderly population (65 years and above) made 40.4 % (*n* = 135) of all papers, while those targeting the population of 55 years and above represented 24.0 % (*n* = 80). The percentage of reviews assessing interventions addressed to the general population including older age groups was 26.3 % (*n* = 88), and those addressed to other age groups encompassing subjects in older age comprised 9.3 % (*n* = 31). From the reviews addressing health promotion interventions, exclusively or in combination with other types, those targeting elderly subjects made up 36.3 % (*n* = 66), and older adults and elderly people represented 25.8 % (*n* = 47) (Table 3). In the reviews addressing primary preventions, these percentages were 42.9 % (*n* = 94) and 23.3 % (*n* = 51), respectively (Table 3).

**Table 3:** Number of systematic reviews retrieved by age and sex categories according to the four general areas of intervention

General area of intervention	Age categories				Sex categories		
	general population^a^	55+	65+	other	both sexes	women	men
health promotion^b^	54	47	66	15	171	9	2
primary prevention^b^	55	51	94	19	205	12	2
screening^b^	14	5	7	8	15	15	4
social support^b^	6	7	18	4	35	0	0

^a^on the condition that it encompassed the elderly population

^b^systematic reviews addressing interventions which were classified as fulfilling the criteria of at least one general area of intervention (either individually or combined with other general area/s)

The overwhelming majority of the systematic reviews analyzed interventions addressed to both sexes (90.1 %, *n* = 301); only 7.8 % (*n* = 26) were related to interventions targeting women and only 2.1 % (*n* = 7) were focused on interventions specific to men. The percentage of reviews targeting both sexes was nearly the same in the studies related to health promotion and primary prevention interventions (94.0 and 93.6 %, respectively; Table 3). There were no gender specific interventions in the reviews classified in the category of social support. The greatest differentiation by sex was seen in systematic reviews classified as including screening interventions; only 44.1 % were focused on both sexes, with 44.1 % targeting women and 11.8 % men (Table 3).

### Year of publication

**Fig. 2:**
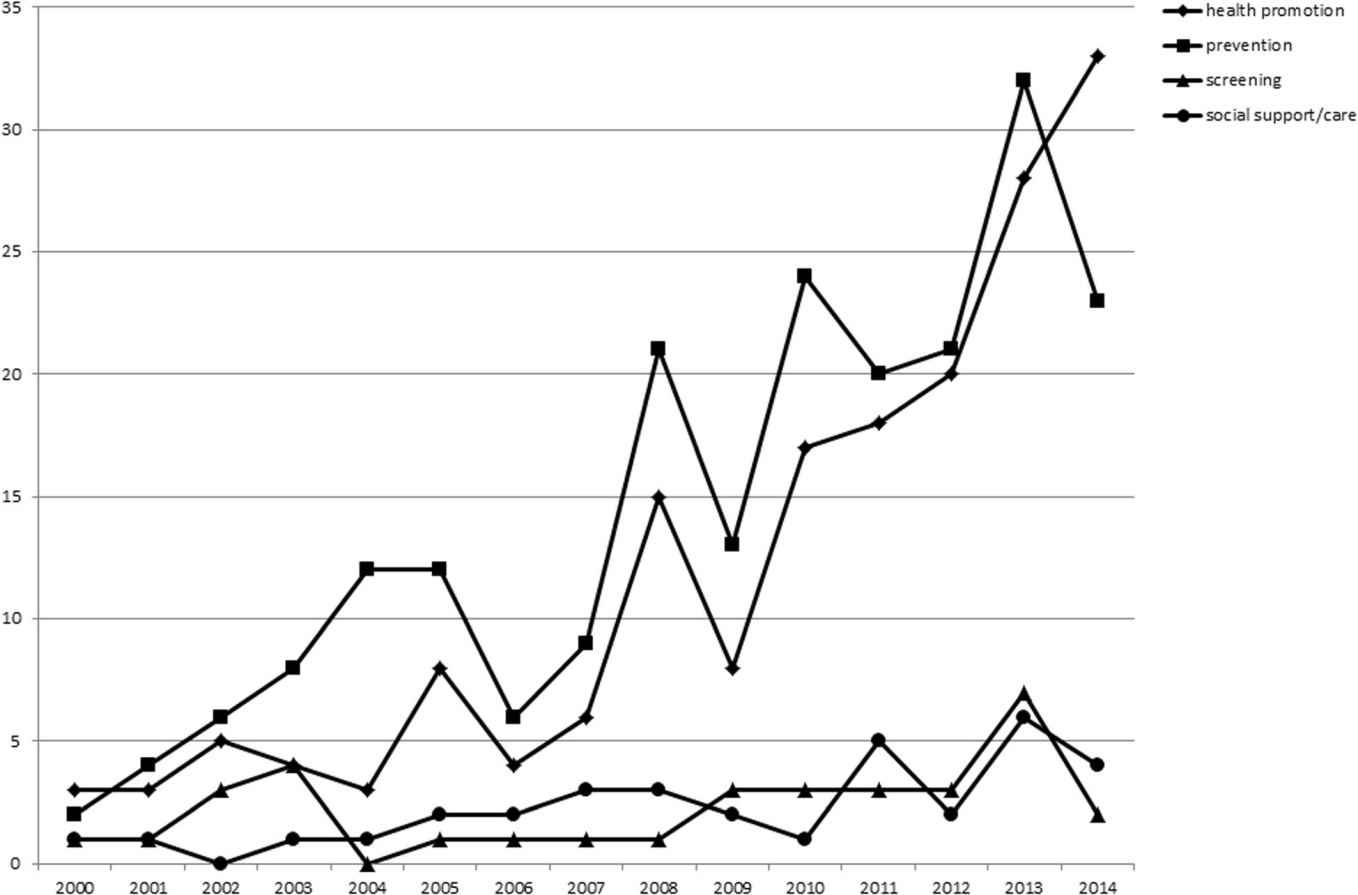
Numbers of systematic reviews retrieved in consecutive years between 2000 and 2014 according to the general area of intervention

The number of systematic reviews corresponding with the inclusion criteria increased steadily from 2000, reaching the highest values in 2013 and 2014. The number of records retrieved from 2015 is relatively low; however, this is due to the fact that the search only included the first 4 months of the year. The number of all systematic reviews retrieved increased from 3 in 2000 to 48 in 2014. The trend was also observed for systematic reviews related to general areas of intervention (Fig. 2). The distribution of systematic reviews according to detailed categories of general areas of interventions and year of publication is presented in Table 4.

**Table 4:** Number of systematic reviews retrieved published between January 2000 and April 2015 according to exclusive categories of interventions

Year	HP	PP	SCR	SS	HP & PP	HP & SS	HP & SCR	HP & PP & SS	PP & SCR	PP & SS	HP & PP & SCR	HP & PP & SCR & SS	Total
2000	1	0	0	0	0	0	0	1	0	0	1	0	3
2001	0	1	1	0	2	0	0	1	0	0	0	0	5
2002	2	4	2	0	2	0	1	0	0	0	0	0	11
2003	2	2	1	0	1	0	0	1	3	0	0	0	10
2004	0	8	0	0	3	0	0	0	0	1	0	0	12
2005	1	5	0	0	5	0	0	1	0	0	0	1	13
2006	1	3	1	1	2	0	0	1	0	0	0	0	9
2007	1	2	0	1	4	0	0	1	1	1	0	0	11
2008	5	10	0	0	7	0	0	3	1	0	0	0	26
2009	2	8	3	1	5	1	0	0	0	0	0	0	20
2010	5	13	2	0	10	0	1	1	0	0	0	0	32
2011	8	8	3	1	8	0	0	2	0	2	0	0	32
2012	12	13	3	2	8	0	0	0	0	0	0	0	38
2013	11	19	4	0	10	5	1	1	2	0	0	0	53
2014	20	13	2	0	9	3	0	1	0	0	0	0	48
2015	4	3	0	0	3	0	0	0	0	1	0	0	11
Total	75	112	22	6	79	9	3	14	7	5	1	1	334

*Abbreviations*: *HP* health promotion, *PP* primary prevention, *SCR* screening, *SS* social support

### Targeted areas of interventions

**Fig. 3:**
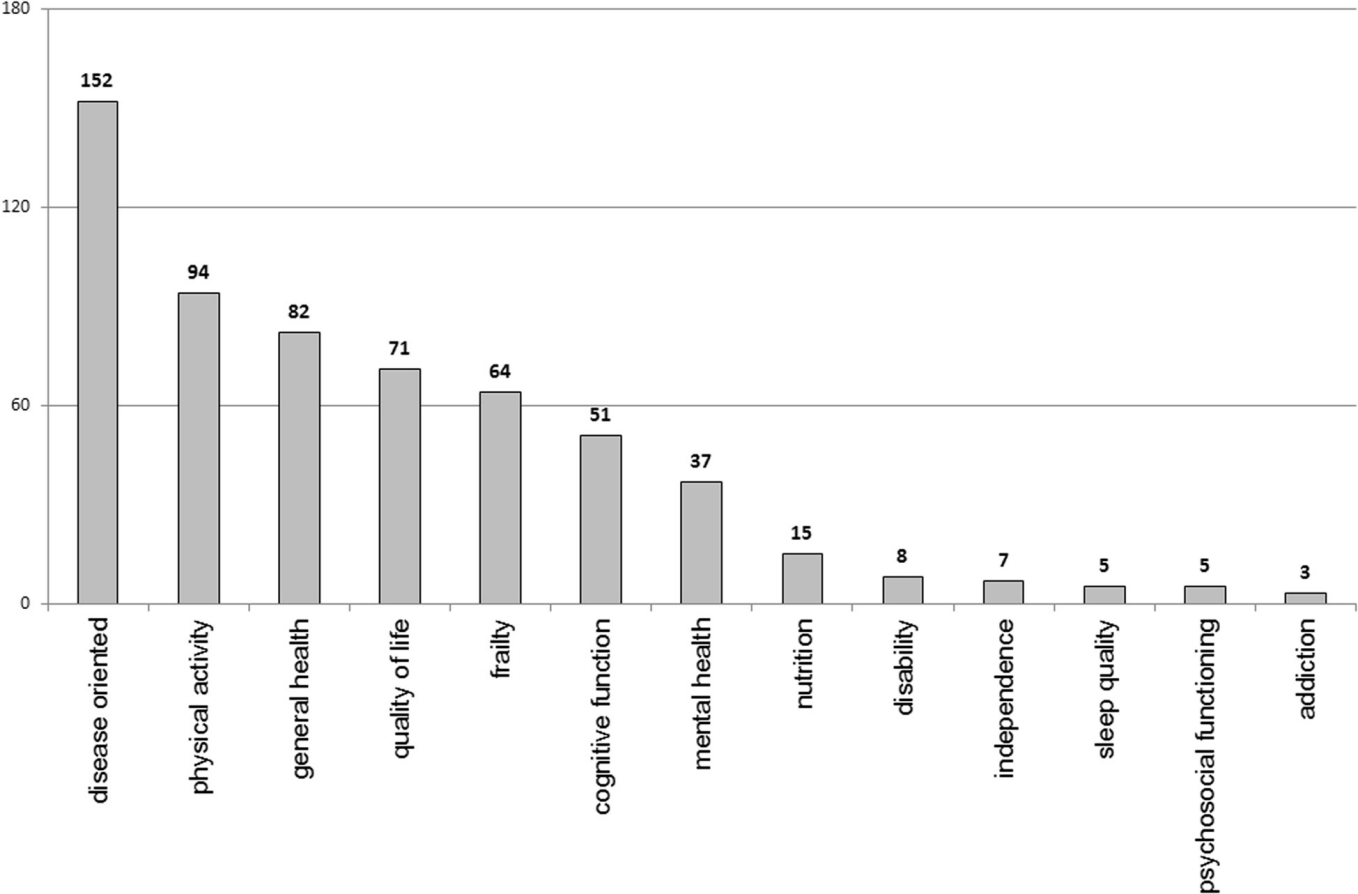
Numbers of systematic reviews retrieved classified by specific target areas of interventions

An initial list of key target problems was established on the basis of the areas targeted by health promotion and related interventions. It was further amended with issues identified in the systematic reviews. The numbers of systematic reviews which could be assigned to specific areas are shown in Fig. 3. As a single systematic review could be assigned to several areas, the total exceeds the number of reviews retrieved.

In the systematic reviews, the three most frequent target areas of interventions were specific diseases (disease-oriented, *n* = 152), physical activity (*n* = 94) and general health (*n* = 82). Other common target areas were quality of life (*n* = 71), frailty (*n* = 64), cognitive function (*n* = 51) and mental health (37). The highest number of systematic reviews addressing disease-oriented interventions is related to the fact that 219 of the 334 reviews addressed the general area of primary prevention. Details of the distribution of interventions according to key target problems with regard to the four general areas and considering the exclusive categories established on the basis of individual and combined areas are presented in Table 5.

**Table 5:** Frequencies of interventions targeted at key problems classified according to the general areas

Target area	HP^a^	PP^a^	SCR^a^	SS^a^	HP^b^	PP^b^	SCR^b^	SS^b^	HP & PP	HP & SCR	HP & SS	PP & SCR	PP & SS	HP & PP & SCR	HP & PP & SS	HP & PP & SCR & SS
disease oriented	65	106	29	15	16	57	21	2	35	3	4	4	3	1	6	0
physical activity	68	59	2	3	35	26	0	0	29	0	0	0	0	1	2	1
general health	71	47	2	12	30	9	0	1	30	0	4	0	1	1	5	1
quality of life	60	37	3	15	26	8	1	2	20	0	5	0	0	1	7	1
frailty	21	54	2	3	7	40	1	1	12	0	1	1	0	0	1	0
cognitive function	36	35	0	5	14	14	0	0	18	0	2	0	1	0	2	0
mental health	20	24	5	6	10	11	1	2	6	0	0	2	1	1	2	1
nutrition	14	7	0	3	6	1	0	0	5	0	2	0	0	0	1	0
disability	5	4	1	2	2	1	1	1	2	0	0	0	0	0	1	0
independence	5	5	0	1	1	2	0	0	3	0	1	0	0	0	0	0
sleep quality	5	3	0	0	2	0	0	0	3	0	0	0	0	0	0	0
psychosocial functioning	3	3	0	4	0	1	0	1	0	0	1	0	0	0	2	0
addiction	3	1	0	0	2	0	0	0	1	0	0	0	0	0	0	0

Categories: SCR&SS, HP&SCR&SS and PP&SCR&SS were not included in the table due to 0 frequencies

*Abbreviations*: *HP* health promotion, *PP* primary prevention, *SCR* screening, *SS* social support

^a^systematic reviews addressing interventions which were classified as fulfilling the criteria of at least one general area of intervention (either individually or combined with other general area/s)

^b^systematic reviews addressing interventions which were classified exclusively as belonging to one general area of intervention

From the 75 systematic reviews assessing the interventions classified exclusively to health promotion, the most numerous target areas of interventions included physical activity (*n* = 35), general health (*n* = 30) and quality of life (*n* = 26). It should be stressed that health promotion interventions were also undertaken relatively frequently in relation to specific diseases (*n* = 16). Within the systematic reviews classified exclusively to primary prevention (*n* = 112), more than half (*n* = 57) were focused on disease-oriented interventions, 40 were related to frailty and 26 to physical activity as a preventive measure. From 22 reviews focused exclusively on screening interventions, 21 were categorized as disease-oriented, which is understandable considering the main aim of such measures.

### Classification of health promotion interventions

**Fig. 4:**
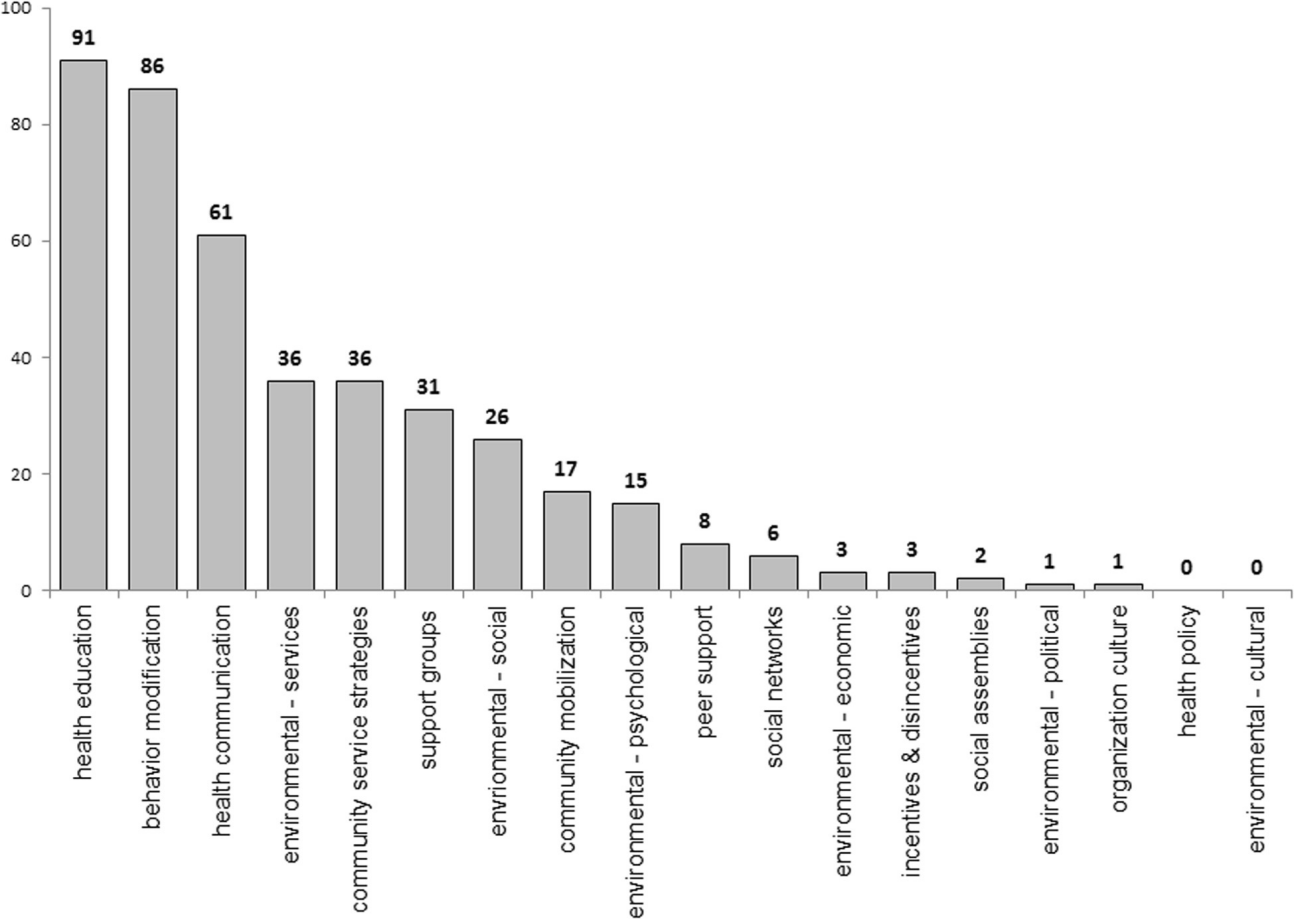
Numbers of systematic reviews retrieved analyzing health promotion interventions classified according to the McKenzie et al. taxonomy

The systematic reviews which covered interventions classified in the domain of health promotion were also classified according to the typology described by McKenzie et al. [13]. The most frequent types of interventions in this cluster of systematic reviews were health education (49.2 %, *n* = 91), behavior modification activities (46.5 %, *n* = 86), and health communication (33.0 %, *n* = 61) (Fig. 4). Less frequent types of interventions encompassed environmental changes related to services modification (19.5 %, *n* = 36), strategies focused on services available in the community (19.5 %, *n* = 36), support groups (16.8 %, *n* = 31) and environmental changes related to the social context (14.1 %, *n* = 26). Other interventions occurred with a frequency below 10 %. No systematic reviews were identified in the domain of health policy and environmental changes in relation to cultural aspects.

## Discussion

The scoping review reported in this paper was carried out with the aim of obtaining a view of the landscape of interventions undertaken within health promotion and related fields in relation to older adults and elderly audiences. Aside from obtaining a broader view of the domain, the results of the scoping review may be further used to guide efforts to identify the types of health promotion interventions, which are actually effective in these specific groups. Finally, the results obtained may be of service for identifying gaps in secondary evidence and future areas of analysis. This study is one of the first efforts aimed at describing the spectrum of health promotion and related interventions targeting health of elderly persons and older adults.

The number of systematic reviews retrieved for consecutive years in the period included in the study increased steadily from 2000. It is understandable when we consider the maturation of the evidence-based public health (EBPH) approach during the recent decades [18, 19]. The original definitions of EBPH were formulated in the late 1990s [20–22]. As well as accepting the need for the evidence-based approach to public health interventions, it also meant that earlier methods used in evidence-based medicine could be applied to some extent in public health. A clear formulation of recommendations for systematic reviews in the areas of public health and health promotion was published in 2007 [23].

Of the general areas of interventions, primary prevention was analyzed the most frequently in the systematic reviews retrieved (65.6 %). The interventions, which could be classified as health promotion activities, were less frequent (54.5 %). Social support and screening interventions were significantly less frequent than the two first areas of interventions and occurred with a similar frequency (10.2 and 10.5 %, respectively). The classification of general areas of interventions was challenging due to the fact that as many as 35.6 % of the systematic reviews were related to interventions stemming from two or more areas. As distinguishing the four general areas of interventions was an arbitrary decision originating from the attempt to describe the scope of actions aimed at the maintenance and improvement of the health of older adults and elderly populations, the interpretation of the proportions between these areas is rather difficult. However, it should be stressed that although systematic reviews analyzing isolated primary prevention interventions were the most frequent category (33.5 %), combined health promotion and primary prevention activities were the second (23.7 %) and isolated health promotion interventions were the third most frequent category (22.5 %). The high number of systematic reviews related to the combined health promotion and primary prevention activities reflects the difficulties with the conceptual separation of both domains [24].

The scoping review aimed to identify health promotion and related interventions addressed to older adults and elderly populations. As a consequence, interventions addressed to the general or other populations, which also included these age groups, were also included in the search strategy. Finally, 40.4 % of the studies were focused on elderly individuals only (65 years and above) and 24.0 % on populations aged at least 55 years. The remaining 35.6 % of the reviews were focused on interventions not addressed specifically to older adults or elderly people, but to population which include them.

The study reported in this paper suffered from several limitations, which were either related to problems with defining specific areas of interventions or to simplifications and rigid assumptions accepted from the start for pragmatic reasons. First of all, it should be underlined that the scoping review did not include the analysis of the effectiveness of types of interventions differentiated in the classification process. The aim of the assessment of the effectiveness was treated as a criterion for inclusion of systematic reviews and/or meta-analyses in the scoping review. So, the results presented here provide a view of the domain but cannot yet be used for formulating policy recommendations for health promotion and related types of interventions which are feasible in elderly persons and older adults. Further analysis is required, supposedly according to the dimensions of interventions’ classification described in this paper.

Furthermore, the authors assumed that classifications made in databases searched and the proposed keywords may be a potential source of ambiguity in the interpretation of the search results. Thus, after applying the search strategy, the systematic reviews were classified from the beginning on the basis of definitions developed and accepted in the study.

Four general areas of interventions were selected arbitrarily based on the general approach aiming to analyze the effectiveness of interventions addressed to healthy or presumably healthy subjects in target audiences. This resulted in the exclusion of papers which reported the effectiveness of therapeutic, rehabilitation or interventions higher than the primary level of disease prevention. The only exemption from this rule was the inclusion of screening procedures.

Additionally, the study included systematic reviews which analyzed interventions belonging to the four general areas and addressing patients with specific medical conditions, but not aimed at these conditions as such but rather at the patient’s general health status.

All these assumptions may be seen as being oversimplified, especially when considering the potential difficulties with indicating health promotion interventions which are not specific to medical conditions occurring in the target audiences of elderly people or older adults.

Another issue which may be perceived as a limitation in this study is the arbitrary assumption of a set of definitions used to describe and classify the papers identified in the search (Additional file 1). The choice or formulation of the definitions was mainly guided by the aim to provide a clear differentiation between existing concepts and categories. The process was carried out on the basis of existing literature and a consensus was sought within the authors’ team.

Finally, on a general level, using secondary evidence available as systematic reviews and/or meta-analyses of the effectiveness of interventions belonging to the areas of interest may be a limitation on obtaining a broader view of the domain. Although further evidence may be available in other sources, its extraction is likely to be demanding and may not even be possible within the framework of this scoping review. In this stage, the aim of defining interventions assessed for their effectiveness in specific age groups justified such strategy.

## Conclusions

Primary prevention measures, used alone or in combination with health promotion interventions, prevail among interventions analyzed in systematic reviews for their effectiveness in populations of elderly people and older adults or general audiences encompassing these age groups. Combined interventions constitute at least one third of all interventions identified in the search. A considerable part of interventions belonging to the four general areas were addressed to general or other populations encompassing older adults and/or elderly individuals. Finally, of the types of health promotion interventions, those classified as health education, behavior modification and health communication were the most frequently analyzed in systematic reviews retrieved.

## Abbreviations

EBPH, evidence-based public health; HP, health promotion; PP, primary prevention; SCR, screening; SS, social support

## Additional files

Additional file 1: Definitions and relevant references used by the authors to describe the studies retrieved. (DOCX 19 kb) Additional file 2: PRISMA Checklist for systematic reviews. (DOCX 27 kb) Additional file 3: List of systematic reviews and/or meta-analyses included in the scoping review with the classification results. (DOCX 60 kb)
