# Supplementary material for: Scoping review of health promotion and disease prevention interventions addressed to elderly people
Source: BMC Health Serv Res. 2016 Sep 5;16(Suppl 5):278. doi: 10.1186/s12913-016-1521-4 (PMC5016725; doi:10.1186/s12913-016-1521-4)
Supplement: Additional file 1: — Definitions and relevant references used by the authors to describe the studies retrieved. (DOCX 19 kb) [file 12913_2016_1521_MOESM1_ESM.docx]

Additional file 1

# Definitions used in the process of classification of retrieved systematic reviews and/or meta-analyses.

**Behaviour modification activities**

Techniques intended to help those in the priority population experience a change in behaviour, often used in intrapersonal-level interventions.

*Source: adapted from [1]*

**Buddy System**

It is a support group consisting of two persons which can take two forms; in first two individuals support each other, in the other form, only one of the two is trying two change a behaviour.

*Source: adapted from [1]*

**Community advocacy**

A process in which the people of the community become involved in the institutions and decisions that will have an impact on their lives. It has potential for creating more support, keeping people informed, influencing decisions, activating nonparticipants, improving service, and making people, plans, and programs more responsive.

*Source: [2]*

**Community building**

An orientation to community that stresses the community assets and shared identity, whether or not task-oriented organizing takes place.

*Sources: [3]*

**Community mobilization**

Community mobilization strategies involve helping communities identify and take action on shared concerns using participatory decision making, and include such methods as empowerment. They include two main types of strategies: community organization and community building, and community advocacy.

*Source: adapted from [1]*

**Community organization**

Process to which community groups are helped to identify common problems or goals, mobilize resources, and in other ways develop and implement strategies for reaching the goals they have collectively set.

*Sources: [3]*

**Disability**

An umbrella term for impairments, activity limitations and participation restrictions. It denotes the negative aspects of the interaction between an individual (with a health condition) and that individual’s contextual factors (environmental and personal factors).

*Source: [4]*

**Disease prevention**

Measures aimed at precluding occurrence of disease or avoiding or slowing down its consequences in persons in whom disease occurred.

*Source: adapted from [5]*

**Disincentive**

Inducement which are negatively valued by the target population and are to be intentionally avoided.

*Source: [6]*

**Effectiveness**

A measure of the extent to which a specific intervention, procedure, regimen, or service, when deployed in the field in routine circumstances, does what it is intended to do for a specified population. In the health field, it is a measure of output from those health services that contribute towards reducing the dimension of a problem or improving an unsatisfactory situation

*Source: [7]*

**Elderly**

In this report, people at least 65 years old

**Environmental change strategies**

Strategies aimed at changing the physical and socio-political environments, providing opportunities, support, and cues to help people develop healthier behaviours complementing individual-level programs and usually more permanent

*Source: adapted from [8]*

**Health communication**

Health communication means informing, influencing, and motivating audiences about important health issues.

*Source: [9]*

**Health education**

Consciously constructed opportunities for learning involving some form of communication designed to improve health literacy, including improving knowledge, and developing life skills which are conducive to individual and community health

*Source: [5]*

**Health policy**

A formal statement or procedure within institutions (notably government) which defines priorities and the parameters for action in response to health needs, available resources and other political pressures.

*Source: [5]*

Health policies/enforcement strategies include executive orders, laws, ordinances, judicial decisions, policies, regulations, rules and position statements.

*Source: [1]*

**Health promotion**

The actions undertaken in order to enable people to increase control over and to improve their health addressed both to individuals as well as to communities and populations.

In the context of the scoping report, health promotion interventions should have been aimed at health determinants as primary target.

*Source: [10]*

**Health-related community service strategies**

Strategies based on reduction of barriers to obtaining services, tests, treatments, or care to improve the health of those in the priority population, e.g. completing low cost flu shots or child immunizations, providing clinical screenings, or providing professional health checkups and examinations.

*Sources: Adapted from [1].*

**Incentive**

It is an anticipated positive or desirable reward designed to influence the performance of an individual or group. An incentive can increase the perceived value of an activity, motivate people to get involved, encourage health service use behaviour, encourage compliance with professional health advice, remind program participants of their commitment to and goals for behaviour change, promote short-term behaviour change, and maintain behaviour change over time.

*Source: adapted and combined from [6] and [1]*

**Intervention**

An intervention comprises an action or programme that aims to bring about identifiable outcomes.

*Source: [11]*

**Older adults**

In this report, population in age of 55-64 years

**Organizational culture activities**

Activities that affect the norms and traditions that are generated by and linked to an organization.

*Sources: adapted from [1].*

**Primary prevention**

Measures undertaken in order to diminish the risk of or avoid development of disease.

*Source: adapted and combined from [5] and [12].*

**Screening**

Use of specific procedures to help determine if apparently well persons have a disease or are at high risk of having a disease.

*Source: [9].*

**Secondary prevention**

Any intervention strategy intended to reduce the presence of an existing disease in a population, thus preventing further deterioration and early death. Secondary prevention is concerned with early detection and prompt treatment of disease.

*Source: adapted [9].*

**Social assembly**

Bringing together people who may be confronting similar problems for the purpose of purely social interaction not related to the problem which can indirectly help them deal with the problem.

*Source: adapted from [1].*

**Support group**

A group of people with common experiences and concerns who provide emotional and moral support for one another.

*Source: [13]*

**Social network**

A type of social intervention based on the web of social relationships that surround an individual and the structural characteristics of that web

Source: *[14]*

The collection of interpersonal ties that people of all ages maintain in varying contexts. While such tries may or may not be supportive, the terms “support network” and “social network” are often used interchangeably.

*Source: [15]*

**Social support**

Support accessible to an individual through social ties to other individuals, groups, and the larger community.

*Source: [16]*

References

1. McKenzie JF, Neiger BL, Thackeray R (eds.). Planning, Implementing & Evaluation Health Promotion Program. A Primer. Six Edition. Boston: Pearson Education Inc; 2013.
2. Checkoway B. Community participation for health promotion: Prescription for public policy. Wellness Perspectives: Research, Theory and Practice. 1989;6(1):18-26.
3. Minkler M, Wallerstein N, Wilson N. Improving health through community organization and community building. In: Glanz K, Rimer BK, Viswanath K, editors. Health behavior and health education. San Francisco: John Wiley & Sons Inc, Jossey-Bass, A Wiley Imprint; 2008.
4. World Health Organization (WHO). The International Classification of Functioning, Disability and Health (ICF). Geneva: WHO; 2001.
5. Nutbeam D. Health promotion glossary. Health Promot Int. 1998;13(4):349-364.
6. Chapman LS. (2005) Incentives: An introduction and story. Part I. Absolute Advantage. 2005;4(7):1-46.http://www.ndworksitewellness.org/docs/step4-designing-wellness-incentive-part1.pdf. Accessed 03 Sept 2015.
7. Wojtczak A. Glossary of Medical Education Terms. December 2000, Revised February 2002. http://www.iime.org/glossary.htm. Accessed 29 Jan 2016.
8. Brownson RC, Haire-Joshu D, Luke DA. Shaping the context of health: A review of environmental and policy approaches in the prevention of chronic diseases. Annu Rev Public Health. 2006;27:341-370.
9. Modeste NN, Tamayose TS. Dictionary of Public Health and Education. Terms and concepts. 2nd edition. John Wiley & Sons, Inc.; 2004.
10. World Health Organization (WHO). The Ottawa Charter for Health Promotion, First International Conference on Health Promotion, Ottawa, 21 November 1986.
11. Rychetnik L, Hawe P, Waters E, Barratt A, Frommer M. A glossary for evidence based public health. J Epidemiol Community Health 2004;58:538-545.
12. The Association of Faculties of Medicine of Canada (AFMC). () Primer on Population Health. A virtual textbook on public health concepts for clinicians. 2001. http://phprimer.afmc.ca. Accessed 10 Sept 2015.
13. Medical Dictionary. An Encyclopaedia Britannica Company. http://www.merriam-webster.com/. Accessed 10 Sept 2015.
14. Institute of Medicine (IOM). Health and behavior: The interplay of biological, behavioral, and societal influence. Washington: DC, National Academy Press (US); 2001.
15. Litwin H. Social Network Type and Morale in Old Age. Gerontologist 2001;41:516-524.
16. Lin N, Ensel WM, Simeone RS, Kuo W. Social support, stressful life events, and illness: A model and an empirical test. J Health Soc Behav. 1979;20(2):108-119.
