# Supplementary material for: Scoping review of health promotion and disease prevention interventions addressed to elderly people
Source: BMC Health Serv Res. 2016 Sep 5;16(Suppl 5):278. doi: 10.1186/s12913-016-1521-4 (PMC5016725; doi:10.1186/s12913-016-1521-4)
Supplement: Additional file 3: — List of systematic reviews and/or meta-analyses included in the scoping review with the classification results. (DOCX 60 kb) [file 12913_2016_1521_MOESM3_ESM.docx]

Additional file 3

| **Authors** | **Year of publication** | **Population, gender** | **General area of intervention** | **Targeted are** | **Type of health promotion intervention (if applicable)** |
| --- | --- | --- | --- | --- | --- |
| Aalbers et al. [1] | 2011 | other population, both genders | health promotion, | general health, | health communication, health education, health-related community services, behaviour modification, support groups, social networks |
| Abbott et al. [2] | 2013 | 65+, both genders | health promotion, | nutrition, | other |
| Agmon et al. [3] | 2014 | 55+, both genders | primary prevention, | disease oriented, physical activity, cognitive function, | na* |
| Aguirre et al. [4] | 2013 | 65+, both genders | primary prevention, | quality of life, cognitive function, | na |
| Anderson et al. [5] | 2014 | 55+, women | health promotion, primary prevention, | physical activity, general health, cognitive function, | behaviour modification, |
| Angevaren et al. [6] | 2008 | 55+, both genders | health promotion, | physical activity, cognitive function, | other |
| Anuruang et al. [7] | 2014 | general population, both genders | health promotion, social support, | disease oriented, general health, quality of life, independence, | health communication, health education, environmental - services, environmental - psychological, community mobilisation, support groups, peer support |
| Ardern et al. [8] | 2013 | 65+, both genders | health promotion, primary prevention, | quality of life, cognitive function, | behaviour modification |
| Arent et al. [9] | 2000 | 55+, both genders | health promotion, | physical activity, general health, quality of life, | behaviour modification |
| Arnold et al. [10] | 2008 | general population, both genders | health promotion, primary prevention, social support, | physical activity, | health communication, health education, environmental - social, behaviour modification |
| Ashworth et al. [11] | 2005 | general population, both genders | health promotion, primary prevention, | disease oriented, physical activity, | other |
| Bandayrel et al. [12] | 2011 | 55+, both genders | health promotion, | general health, quality of life, nutrition, | health communication, health education, health-related community services, behaviour modification, support groups |
| Barlow et al. [13] | 2007 | general population, both genders | health promotion, primary prevention, | disease oriented, frailty, mental health, | health communication, health education, health-related community services |
| Baron et al. [14] | 2010 | general population, both genders | health promotion, screening, | disease oriented, | environmental - services, health-related community services |
| Batchelor et al. [15] | 2010 | general population, both genders | primary prevention, | disease oriented, | na |
| Bemelmans et al. [16] | 2012 | 65+, both genders | social support, | disease oriented, quality of life, frailty, mental health, | na |
| Berzins et al. [17] | 2009 | other population, both genders | social support, | disease oriented, | na |
| Beswick et al. [18] | 2008 | 65+, both genders | health promotion, primary prevention, social support, | disease oriented, general health, quality of life, disability, | health communication, health education, environmental - services, environmental - social, support groups |
| Beyer et al. [19] | 2013 | 65+, both genders | primary prevention, | disease oriented, | na |
| Blake et al. [20] | 2009 | 65+, both genders | primary prevention, | mental health, | na |
| Blankevoort et al. [21] | 2010 | 65+, both genders | health promotion, | disease oriented, physical activity, cognitive function, | behaviour modification |
| Bleakley et al. [22] | 2015 | 65+, both genders | health promotion, | physical activity, cognitive function, | health education, environmental - psychological |
| Blondell et al. [23] | 2014 | other population, both genders | primary prevention, | disease oriented, cognitive function, | na |
| Bodner et al. [24] | 2009 | general population, both genders | health promotion, primary prevention, | addiction, | health communication, health education, behaviour modification |
| Bolam et al. [25] | 2013 | general population, men | primary prevention, | physical activity, | na |
| Bonfill Cosp et al. [26] | 2001 | other population, women | screening, | disease oriented, | na |
| Booth et al. [27] | 2014 | general population, both genders | health promotion, primary prevention, | disease oriented, physical activity, general health, | other |
| Bouman et al. [28] | 2008 | 65+, both genders | health promotion, primary prevention, social support, | disease oriented, general health, frailty, | health education, environmental - services, health-related community services |
| Brown et al. [29] | 2013 | 65+, both genders | health promotion, | physical activity, disability, | health communication, health education |
| Brown et al. [30] | 2012 | general population, both genders | primary prevention, | disease oriented, quality of life, mental health, | na |
| Bullo et al. [31] | 2015 | 65+, both genders | health promotion, primary prevention, | disease oriented, physical activity, general health, quality of life, mental health, | other |
| Cameron et al. [32] | 2010 | 65+, both genders | primary prevention, | disease oriented, frailty, | na |
| Cameron et al. [33] | 2012 | 65+, both genders | primary prevention, | frailty, | na |
| Camilloni et al. [34] | 2013 | other population, both genders | primary prevention, screening, | disease oriented, | na |
| Cardona-Morrell et al. [35] | 2010 | general population, both genders | health promotion, primary prevention, | disease oriented, physical activity, nutrition, | health education, environmental - social, environmental - psychological, health-related community services, behaviour modification |
| Carter et al. [36] | 2001 | 55+, both genders | primary prevention, | frailty, | na |
| Carvalho et al. [37] | 2014 | 55+, both genders | health promotion, | cognitive function, | health education, behaviour modification, |
| Cattan et al. [38] | 2005 | other population, both genders | health promotion, primary prevention, social support, |  | health communication, health education, support groups, peer support, social assemblies, social networks |
| Chang et al. [39] | 2004 | 65+, both genders | primary prevention, | disease oriented, physical activity, frailty, | na |
| Chi et al. [40] | 2012 | 55+, both genders | primary prevention, | physical activity, mental health, | na |
| Chien et al. [41] | 2008 | general population, both genders | health promotion, primary prevention, | disease oriented, quality of life, | na |
| Chin et al. [42] | 2007 | 65+, both genders | social support, | general health, quality of life, | na |
| Chin a Paw et al. [43] | 2008 | general population, both genders | health promotion, primary prevention, | frailty, | other |
| Choi et al. [44] | 2012 | 65+, both genders | social support, | mental health, | na |
| Chou et al. [45] | 2012 | 65+, both genders | health promotion, | physical activity, quality of life, frailty, | other |
| Chou et al. [46] | 2011 | other population, both genders | screening, | disease oriented, disability, | na |
| Clark et al. [47] | 2012 | 55+, both genders | health promotion, | physical activity, | other |
| Clegg et al. [48] | 2012 | 65+, both genders | health promotion, primary prevention, | physical activity, general health, quality of life, frailty, | health communication, health education, environmental - services |
| Clegg et al. [49] | 2014 | 65+, both genders | primary prevention, | disease oriented, | na |
| Clemson et al. [50] | 2008 | 65+, both genders | primary prevention, | frailty, | na |
| Cole et al. [51] | 2004 | other population, both genders | primary prevention, | mental health, | na |
| Cole et al. [52] | 2008 | general population, both genders | primary prevention, | disease oriented, mental health, | na |
| Conn et al. [53] | 2011 | general population, both genders | health promotion, | general health, | health education, behaviour modification |
| Conn et al. [54] | 2003 | general population, both genders | health promotion, | physical activity, | health communication, health education, environmental - social, behaviour modification |
| Conn et al. [55] | 2009 | general population, both genders | health promotion, primary prevention, | physical activity, quality of life, | health education, behaviour modification, support groups |
| Conn et al. [56] | 2002 | 65+, both genders | health promotion, | physical activity, | health communication, health education, environmental - services, community mobilisation, behaviour modification, support groups, peer support |
| Cooper et al. [57] | 2012 | other population, both genders | health promotion, primary prevention, | quality of life, cognitive function, | health communication, health education, environmental - social, environmental - psychological, behaviour modification, support groups |
| Costello et al. [58] | 2008 | 65+, both genders | primary prevention, | frailty, | na |
| Crew et al. [59] | 2015 | 55+, both genders | health promotion, | disease oriented, physical activity, | other |
| Crichton et al. [60] | 2010 | general population, both genders | health promotion, primary prevention, | cognitive function, | health communication, environmental - services |
| Crocker et al. [61] | 2013 | 65+, both genders | health promotion, | general health, quality of life, | other |
| Crocker et al. [62] | 2013 | 55+, both genders | health promotion, primary prevention, social support, | physical activity, | health communication, health education, environmental - services, environmental - social, community mobilisation, behaviour modification, support groups, social networks |
| Cuijpers et al. [63] | 2009 | general population, both genders | primary prevention, | disease oriented, mental health, | na |
| Cusimano et al. [64] | 2008 | 65+, both genders | primary prevention, | frailty, | na |
| Dale et al. [65] | 2008 | general population, both genders | health promotion, primary prevention, | disease oriented, general health, | health communication, health education, environmental - psychological, peer support |
| Daniels et al. [66] | 2008 | 65+, both genders | primary prevention, | physical activity, general health, frailty, nutrition, disability, | na |
| Darvishian et al. [67] | 2014 | general population, both genders | primary prevention, | disease oriented, | na |
| Darvishiana et al. [68] | 2014 | general population, both genders | primary prevention, | disease oriented, | na |
| de Kam et al. [69] | 2009 | 55+, both genders | primary prevention, | physical activity, | na |
| de Niet et al. [70] | 2009 | general population, both genders | health promotion, | disease oriented, sleep quality, | health education, behaviour modification |
| de Oliveira Francisco et al. [71] | 2015 | 55+, both genders | health promotion, | physical activity, general health, | other |
| de Waure et al. [72] | 2013 | other population, both genders | health promotion, primary prevention, | physical activity, mental health, nutrition, | health communication, health education, community mobilisation, behaviour modification |
| DIBardino et al. [73] | 2012 | other population, both genders | primary prevention, | disease oriented, | na |
| Dickens et al. [74] | 2011 | 55+, both genders | primary prevention, social support, | general health, mental health, | na |
| Diep et al. [75] | 2010 | general population, both genders | primary prevention, | disease oriented, | na |
| Dixon et al. [76] | 2007 | 55+, both genders | primary prevention, social support, | disease oriented, | na |
| Djulbegovic et al. [77] | 2010 | 65+, men | screening, | disease oriented, | na |
| Dombrowski et al. [78] | 2010 | general population, both genders | health promotion, primary prevention, | physical activity, general health, nutrition, | health communication, health education, environmental - services, environmental - social, health-related community services, community mobilisation, support groups |
| Eells et al. [79] | 2014 | 65+, both genders | health promotion, | disease oriented, quality of life, | environmental - social, community mobilisation, support groups |
| Elkan et al. [80] | 2001 | 65+, both genders | health promotion, primary prevention, social support, | general health, quality of life, | health communication, health education, health-related community services |
| Elkan et al. [81] | 2000 | 65+, both genders | health promotion, primary prevention, screening, | disease oriented, physical activity, general health, quality of life, mental health, | health communication, health education, environmental - services, environmental - social, environmental - psychological, health-related community services, community mobilisation |
| El-Khoury et al. [82] | 2013 | 65+, both genders | primary prevention, | physical activity, frailty, | na |
| Evans et al. [83] | 2003 | 65+, both genders | health promotion, | general health, quality of life, independence, | other |
| Fairhall et al. [84] | 2011 | 55+, both genders | primary prevention, | physical activity, general health, | na |
| Faucounau et al. [85] | 2010 | 65+, both genders | primary prevention, | disease oriented, cognitive function, | na |
| Felix et al. [86] | 2013 | 55+, both genders | primary prevention, | general health, | na |
| Ferreira et al. [87] | 2012 | 55+, both genders | health promotion, primary prevention, | physical activity, general health, | other |
| Ferroni et al. [88] | 2012 | general population, both genders | screening, | disease oriented, | na |
| Forbes et al. [89] | 2015 | 65+, both genders | primary prevention, social support, | cognitive function, | na |
| Forsman et al. [90] | 2011 | 65+, both genders | health promotion, primary prevention, social support, | disease oriented, quality of life, mental health, | health education, support groups |
| Forsman et al. [91] | 2011 | 65+, both genders | primary prevention, social support, | disease oriented, | na |
| Gagliardi et al. [92] | 2012 | 55+, both genders | primary prevention, | disease oriented, | na |
| Gard et al. [93] | 2014 | general population, both genders | health promotion, primary prevention, | cognitive function, | health communication, health education, health-related community services |
| Gardner et al. [94] | 2013 | other population, women | health promotion, screening, | disease oriented, | health communication, health education, environmental - economic, health-related community services, incentives & disincentives, support groups, peer support |
| Gates et al. [95] | 2013 | 65+, both genders | primary prevention, | cognitive function, | na |
| Gellis et al. [96] | 2012 | 65+, both genders | primary prevention, | disease oriented, | na |
| Geraedts et al. [97] | 2013 | general population, both genders | health promotion, | physical activity, general health, quality of life, | health communication, health education, environmental - services, health-related community services, behaviour modification |
| Gillespie et al. [98] | 2003 | 65+, both genders | primary prevention, | disease oriented, physical activity, frailty, cognitive function, | na |
| Gillespie et al. [99] | 2009 | 65+, both genders | primary prevention, | disease oriented, physical activity, frailty, | na |
| Gillespie et al. [100] | 2013 | other population, both genders | primary prevention, | disease oriented, | na |
| Gine-Garriga et al. [101] | 2014 | 65+, both genders | health promotion, | physical activity, quality of life, frailty, | na |
| Gleeson et al. [102] | 2014 | 55+, both genders | health promotion, primary prevention, | physical activity, frailty, | health communication, environmental - services, support groups |
| Gnoato Zotz et al. [103] | 2014 | 65+, both genders | health promotion, | physical activity, | na |
| Gobbo et al. [104] | 2014 | 55+, both genders | health promotion, | general health, | na |
| Goodwin et al. [105] | 2011 | general population, both genders | primary prevention, | frailty, | na |
| Goodwin et al. [106] | 2014 | 65+, both genders | primary prevention, | frailty, | na |
| Gotzsche et al. [107] | 2013 | general population, women | screening, | disease oriented, | na |
| Gould et al. [108] | 2014 | 65+, both genders | health promotion, | quality of life, cognitive function, mental health, addiction, | health communication, health education, behaviour modification |
| Gregory et al. [109] | 2009 | other population, both genders | primary prevention, | disease oriented, | na |
| Gu et al. [110] | 2008 | 65+, both genders | health promotion, | physical activity, | health communication, health education, behaviour modification |
| Guo et al. [111] | 2013 | 65+, both genders | primary prevention, | frailty, | na |
| Hackshaw et al. [112] | 2003 | 55+, women | screening, | disease oriented, | na |
| Hamilton et al. [113] | 2010 | 55+, women | primary prevention, | physical activity, | na |
| Harling et al. [114] | 2008 | 65+, both genders | primary prevention, | disease oriented, | na |
| Harris et al. [115] | 2011 | general population, women | screening, | disease oriented, | na |
| Hauer et al. [116] | 2006 | 65+, both genders | health promotion, primary prevention, | disease oriented, general health, frailty, disability, | health communication, environmental - social, health-related community services, community mobilisation, support groups, peer support |
| Hayes et al. [117] | 2014 | 55+, men | screening, | disease oriented, | na |
| Healey et al. [118] | 2008 | other population, both genders | primary prevention, | quality of life, frailty, | na |
| Heaven et al. [119] | 2013 | 55+, both genders | health promotion, social support, | general health, quality of life, | behaviour modification, support groups, social assemblies |
| Heyn et al. [120] | 2008 | 65+, both genders | health promotion, | disease oriented, physical activity, cognitive function, | environmental - services, support groups |
| Hill-Westmoreland et al. [121] | 2002 | 65+, both genders | primary prevention, | disease oriented, physical activity, frailty, | na |
| Hobbs et al. [122] | 2013 | 55+, both genders | health promotion, | physical activity, | health communication, health education, behaviour modification |
| Howe et al. [123] | 2011 | 55+, both genders | health promotion, primary prevention, | disease oriented, physical activity, | other |
| Huang et al. [124] | 2005 | 55+, both genders | health promotion, primary prevention, | physical activity, general health, quality of life, | other |
| Huang et al. [125] | 2013 | 65+, both genders | primary prevention, | disease oriented, physical activity, | na |
| Humphrey et al. [126] | 2002 | general population, women | screening, | disease oriented, | na |
| Huss et al. [127] | 2008 | 65+, both genders | health promotion, primary prevention, | general health, frailty, disability, | health education, environmental - services, health-related community services, behaviour modification |
| Ilic et al. [128] | 2011 | general population, men | screening, | disease oriented, | na |
| Ilic et al. [129] | 2013 | general population, men | screening, | disease oriented, quality of life, | na |
| Ishigaki et al. [130] | 2014 | 65+, both genders | primary prevention, | physical activity, frailty, | na |
| Jefferson et al. [131] | 2005 | 65+, both genders | primary prevention, | disease oriented, | na |
| Jefferson et al. [132] | 2010 | 65+, both genders | primary prevention, | disease oriented, | na |
| Joe et al. [133] | 2013 | 55+, both genders | health promotion, primary prevention, | disease oriented, general health, cognitive function, | health communication, health education, behaviour modification |
| Jonker et al. [134] | 2009 | 55+, both genders | health promotion, | disease oriented, physical activity, general health, quality of life, frailty, mental health, nutrition, | health communication, health education, environmental - psychological, behaviour modification |
| Kachouie et al. [135] | 2014 | general population, both genders | health promotion, | general health, quality of life, mental health, | health education, environmental - services, environmental - social, peer support |
| Kelley et al. [136] | 2001 | other population, both genders | health promotion, primary prevention, | general health, | behaviour modification |
| Kelley et al. [137] | 2005 | 55+, both genders | health promotion, primary prevention, | disease oriented, general health, | other |
| Kelley et al. [138] | 2013 | general population, men | health promotion, | disease oriented, | other |
| Kelley et al. [139] | 2002 | 55+, women | health promotion, primary prevention, | disease oriented, physical activity, | other |
| Kelly et al. [140] | 2014 | general population, both genders | health promotion, | cognitive function, | other |
| Kelly et al. [141] | 2014 | general population, both genders | health promotion, | cognitive function, | other |
| Kelly et al. [142] | 2001 | general population, women | health promotion, primary prevention, | physical activity, general health, | environmental - services, health-related community services, behaviour modification |
| Kemmler et al. [143] | 2013 | general population, both genders | primary prevention, | physical activity, | na |
| Kendrick et al. [144] | 2014 | 65+, both genders | primary prevention, | disease oriented, physical activity, frailty, | na |
| Kosse et al. [145] | 2013 | general population, both genders | primary prevention, | frailty, | na |
| Kösters et al. [146] | 2003 | other population, women | primary prevention, screening, | disease oriented, | na |
| Kotwal et al. [147] | 2012 | general population, both genders | primary prevention, | disease oriented, | na |
| Kua et al. [148] | 2007 | 55+, both genders | health promotion, primary prevention, | independence, | health communication, health education, environmental - psychological, behaviour modification |
| Kueider et al. [149] | 2012 | general population, both genders | health promotion, primary prevention, | cognitive function, | behaviour modification |
| Lai et al. [150] | 2010 | 65+, women | health promotion, primary prevention, | disease oriented, physical activity, quality of life, nutrition, | health education, environmental - services, health-related community services, behaviour modification |
| Lara et al. [151] | 2014 | 55+, both genders | health promotion, | general health, nutrition, | health education, behaviour modification |
| Lau et al. [152] | 2012 | 55+, both genders | primary prevention, | disease oriented, | na |
| Law et al. [153] | 2014 | general population, both genders | health promotion, | cognitive function, | other |
| Lee et al. [154] | 2010 | 55+, both genders | health promotion, primary prevention, | general health, | health education, behaviour modification |
| Lee et al. [155] | 2008 | general population, both genders | primary prevention, | disease oriented, physical activity, | na |
| Lee et al. [156] | 2012 | 55+, both genders | primary prevention, | cognitive function, | na |
| Legg et al. [157] | 2006 | 55+, both genders | social support, | disability, | na |
| Legler et al. [158] | 2002 | 55+, women | health promotion, screening, | disease oriented, | health communication, environmental - economic, environmental - services, health-related community services, community mobilisation |
| Leung et al. [159] | 2011 | 65+, both genders | health promotion, primary prevention, | disease oriented, physical activity, | na |
| Leung et al. [160] | 2002 | other population, women | screening, | disease oriented, | na |
| Liebel et al. [161] | 2009 | 65+, both genders | health promotion, social support, | frailty, | health communication, health education, environmental - services, health-related community services |
| Littbrand et al. [162] | 2011 | 65+, both genders | health promotion, primary prevention, | disease oriented, quality of life, cognitive function, | health education, behaviour modification, |
| Liu et al. [163] | 2014 | 65+, both genders | health promotion, primary prevention, social support, | nutrition, | health education, environmental - services, environmental - social |
| Liu et al. [164] | 2013 | 65+, women | health promotion, primary prevention, | disease oriented, | health communication, health education, behaviour modification |
| Liu et al. [165] | 2009 | general population, both genders | health promotion, primary prevention, | physical activity, | behaviour modification |
| Liu et al. [166] | 2011 | general population, men | health promotion, primary prevention, | disease oriented, | health-related community services |
| Lock et al. [167] | 2006 | other population, both genders | health promotion, primary prevention, | disease oriented, | other |
| Logghe et al. [168] | 2010 | 55+, both genders | primary prevention, | frailty, independence, | na |
| Lopopolo et al. [169] | 2006 | 65+, both genders | health promotion, | general health, quality of life, | behaviour modification, support groups |
| Low et al. [170] | 2009 | 65+, both genders | primary prevention, | general health, | na |
| Luna et al. [171] | 2010 | other population, both genders | primary prevention, | disease oriented, | na |
| Manafo et al. [172] | 2012 | 55+, both genders | health promotion, | general health, | health communication, health education, environmental - social, social networks |
| Mandelblatt et al. [173] | 2003 | general population, women | primary prevention, screening, | disease oriented, | na |
| Manzoli et al. [174] | 2012 | 65+, both genders | primary prevention, | disease oriented, | na |
| March et al. [175] | 2015 | general population, both genders | health promotion, primary prevention, | disease oriented, general health, quality of life, mental health, | health communication, health education, environmental - services, environmental - social, community mobilisation, behaviour modification, peer support |
| Markle-Reid et al. [176] | 2006 | 65+, both genders | health promotion, primary prevention, social support, | general health, | health communication, health education, environmental - services, health-related community services, behaviour modification, support groups |
| Marks et al. [177] | 2004 | 55+, both genders | primary prevention, | frailty, | na |
| Marmot et al. [178] | 2012 | general population, women | screening, | disease oriented, | na |
| Marques et al. [179] | 2012 | 65+, both genders | primary prevention, | physical activity, | na |
| Marshall et al. [180] | 2013 | 65+, both genders | health promotion, social support, | general health, nutrition, | environmental - social, behaviour modification |
| Martin et al. [181] | 2011 | 65+, both genders | health promotion, | cognitive function, | health education, behaviour modification, support groups |
| Martin et al. [182] | 2013 | 65+, both genders | primary prevention, | frailty, | na |
| Martinez et al. [183] | 2015 | 55+, both genders | primary prevention, | disease oriented, cognitive function, | na |
| Martyn-St James et al. [184] | 2008 | 55+, women | primary prevention, | disease oriented, | na |
| Massat et al. [185] | 2013 | general population, both genders | primary prevention, screening, | disease oriented, | na |
| Maujean et al. [186] | 2014 | 55+, both genders | health promotion, primary prevention, | general health, | health education, behaviour modification |
| McClure et al. [187] | 2005 | 65+, both genders | health promotion, primary prevention, | disease oriented, physical activity, frailty, | health communication, health education, environmental - services, health-related community services, community mobilisation |
| McDaid et al. [188] | 2011 | 55+, both genders | health promotion, primary prevention, social support, | quality of life, mental health, | health communication, health education, environmental - services, community mobilisation, behaviour modification, incentives & disincentives, support groups |
| McDermott et al. [189] | 2013 | general population, both genders | primary prevention, | disease oriented, quality of life, | na |
| McGrath et al. [190] | 2009 | 65+, both genders | health promotion, primary prevention, | general health, | health education, behaviour modification |
| McNamara et al. [191] | 2013 | 55+, both genders | health promotion, primary prevention, | disease oriented, | behaviour modification |
| McPhate et al. [192] | 2013 | 55+, both genders | health promotion, primary prevention, | physical activity, frailty, | health education, health-related community services |
| Michael et al. [193] | 2010 | 65+, both genders | primary prevention, | frailty, | na |
| Michiels et al. [194] | 2011 | 65+, both genders | primary prevention, | disease oriented, | na |
| Michikawa et al. [195] | 2009 | 65+, both genders | screening, | frailty, | na |
| Miller et al. [196] | 2014 | general population, both genders | health promotion, | physical activity, | other |
| Miller et al. [197] | 2013 | general population, both genders | health promotion, | disease oriented, physical activity, general health, | behaviour modification |
| Moayyeri A et al. [198] | 2008 | general population, both genders | health promotion, primary prevention, | disease oriented, frailty, | health education, behaviour modification |
| Moberley et al. [199] | 2013 | 55+, both genders | primary prevention, | disease oriented, | na |
| Montgomery et al. [200] | 2004 | 65+, both genders | health promotion, primary prevention, | cognitive function, sleep quality, | environmental - psychological, behaviour modification |
| Montgomery et al. [201] | 2003 | 55+, both genders | health promotion, primary prevention, | disease oriented, sleep quality, | health communication, environmental - psychological, health-related community services, support groups |
| Moran et al. [202] | 2013 | general population, both genders | screening, | disease oriented, | na |
| Moreland et al. [203] | 2003 | 65+, both genders | primary prevention, screening, | frailty, | na |
| Morris et al. [204] | 2014 | general population, both genders | health promotion, social support, | quality of life, | health communication, environmental - social, community mobilisation, support groups, social networks |
| Müller-Riemenschneider et al. [205] | 2008 | general population, both genders | health promotion, | physical activity, | health communication, health education, behaviour modification |
| Mulligan et al. [206] | 2014 | 55+, both genders | primary prevention, | independence, | na |
| Murphy et al. [207] | 2007 | general population, both genders | health promotion, primary prevention, | disease oriented, general health, | other |
| Naqvi et al. [208] | 2013 | 65+, both genders | primary prevention, | cognitive function, | na |
| Ndiaye et al. [209] | 2005 | general population, both genders | primary prevention, | disease oriented, | na |
| Neidrick et al. [210] | 2012 | general population, both genders | health promotion, | physical activity, | health communication, health education, behaviour modification |
| Nelson et al. [211] | 2009 | 65+, women | screening, | disease oriented, | na |
| Netz et al. [212] | 2005 | 55+, both genders | health promotion, | general health, quality of life, mental health, | na |
| Neyens et al. [213] | 2011 | 65+, both genders | primary prevention, | disease oriented, | na |
| Nichol et al. [214] | 2005 | 65+, both genders | primary prevention, | disease oriented, | na |
| Nickson et al. [215] | 2012 | general population, women | screening, | disease oriented, | na |
| Nikander et al. [216] | 2010 | general population, both genders | health promotion, | general health, | behaviour modification |
| O’Connor et al. [217] | 2009 | general population, both genders | screening, | disease oriented, mental health, | na |
| O’Hare et al. [218] | 2013 | 65+, both genders | primary prevention, | physical activity, quality of life, frailty, | na |
| Öhman et al. [219] | 2014 | 65+, both genders | health promotion, | cognitive function, | other |
| Oliver et al. [220] | 2006 | 65+, both genders | primary prevention, | disease oriented, physical activity, frailty, | na |
| Ooi et al. [221] | 2011 | 55+, both genders | health promotion, primary prevention, | cognitive function, | health-related community services |
| Orr et al. [222] | 2008 | 55+, both genders | health promotion, | disease oriented, general health, frailty, | environmental - services, environmental - social, behaviour modification, support groups |
| Oyama et al. [223] | 2008 | 55+, both genders | primary prevention, screening, | mental health, | na |
| Paap et al. [224] | 2010 | other population, women | screening, | disease oriented, | na |
| Pace et al. [225] | 2014 | general population, women | screening, | disease oriented, | na |
| Park et al. [226] | 2014 | 65+, both genders | health promotion, | disease oriented, quality of life, | other |
| Parker et al. [227] | 2006 | 65+, both genders | primary prevention, | disease oriented, | na |
| Parker et al. [228] | 2004 | 65+, both genders | primary prevention, |  | na |
| Passos et al. [229] | 2012 | general population, both genders | health promotion, | disease oriented, physical activity, sleep quality, | behaviour modification |
| Patel et al. [230] | 2012 | 55+, both genders | health promotion, | general health, quality of life, | health communication, behaviour modification |
| Perry et al. [231] | 2010 | general population, both genders | health promotion, primary prevention, social support, | disease oriented, quality of life, cognitive function, | health education, health-related community services, behaviour modification |
| Peterson et al. [232] | 2010 | 65+, both genders | health promotion, | physical activity, | other |
| Peterson et al. [233] | 2011 | 55+, both genders | primary prevention, | general health, frailty, | na |
| Petridou et al. [234] | 2009 | 65+, both genders | primary prevention, | frailty, | na |
| Pitkälä et al. [235] | 2013 | 65+, both genders | health promotion, primary prevention, | disease oriented, physical activity, | other |
| Plawecki et al. [236] | 2010 | general population, both genders | health promotion, primary prevention, | general health, | health communication, health education, behaviour modification |
| Ploeg et al. [237] | 2005 | 65+, both genders | health promotion, primary prevention, screening, social support, | physical activity, general health, quality of life, mental health, | health communication, health education, environmental - economic, environmental - services, environmental - social, health-related community services, behaviour modification, incentives & disincentives |
| Polisena et al. [238] | 2010 | 65+, both genders | health promotion, primary prevention, | disease oriented, quality of life, | health communication, health education, environmental - services, health-related community services |
| Potter et al. [239] | 2011 | 55+, both genders | primary prevention, | physical activity, mental health, | na |
| Primack et al. [240] | 2012 | general population, both genders | health promotion, | disease oriented, general health, | behaviour modification |
| Rand et al. [241] | 2011 | 55+, both genders | primary prevention, | physical activity, | na |
| Raymond et al. [242] | 2013 | 65+, both genders | health promotion, | physical activity, quality of life, | other |
| Reeder et al. [243] | 2013 | other population, both genders | health promotion, social support, | quality of life, | health education, environmental - services, behaviour modification |
| Reijnders et al. [244] | 2013 | general population, both genders | health promotion, | disease oriented, cognitive function, | health education, |
| Roberts et al. [245] | 2013 | 65+, both genders | screening, | disease oriented, | na |
| Robertson et al. [246] | 2002 | 65+, both genders | primary prevention, | disease oriented, frailty, | na |
| Rodrigues et al. [247] | 2014 | other population, both genders | health promotion, primary prevention, | general health, | na |
| Roets-Merken et al. [248] | 2015 | 55+, both genders | health promotion, primary prevention, | disease oriented, physical activity, quality of life, cognitive function, | health communication, health education, environmental - psychological, behaviour modification |
| Rogers et al. [249] | 2009 | 55+, both genders | health promotion, primary prevention, | physical activity, quality of life, mental health, | other |
| Ross et al. [250] | 2013 | general population, both genders | health promotion, primary prevention, | physical activity, cognitive function, independence, | health education, behaviour modification |
| Rubenstein et al. [251] | 2006 | 65+, both genders | primary prevention, | physical activity, general health, | na |
| Rydwik et al. [252] | 2004 | 65+, both genders | primary prevention, | physical activity, general health, | na |
| Ryhanen et al. [253] | 2010 | 65+, women | primary prevention, | disease oriented, | na |
| Santesso et al. [254] | 2014 | 65+, both genders | primary prevention, | disease oriented, | na |
| Sawka et al. [255] | 2005 | 65+, both genders | primary prevention, | frailty, | na |
| Sawka et al. [256] | 2007 | 65+, both genders | primary prevention, | frailty, | na |
| Schoene et al. [257] | 2014 | other population, both genders | primary prevention, | frailty, cognitive function, | na |
| Schroeder et al. [258] | 2004 | general population, both genders | primary prevention, social support, | disease oriented, | na |
| Schwenk et al. [259] | 2013 | 65+, both genders | health promotion, primary prevention, | physical activity, frailty, | other |
| Sharp et al. [260] | 2014 | 65+, both genders | health promotion, | disease oriented, physical activity, quality of life, | other |
| Sherrington et al. [261] | 2008 | general population, both genders | primary prevention, | frailty, | na |
| Siervo et al. [262] | 2011 | general population, both genders | health promotion, primary prevention, | cognitive function, | health education, behaviour modification |
| Sivan et al. [263] | 2010 | 65+, both genders | primary prevention, | frailty, | na |
| Sjösten et al. [264] | 2008 | 65+, both genders | health promotion, primary prevention, | disease oriented, independence, | health communication, health education, environmental - services, health-related community services, community mobilisation |
| Skelton et al. [265] | 2013 | other population, both genders | health promotion, social support, | disease oriented, general health, | other |
| Smeeth et al. [266] | 2006 | 65+, both genders | screening, | disease oriented, | na |
| Smits et al. [267] | 2007 | general population, both genders | health promotion, primary prevention, social support, | disease oriented, quality of life, cognitive function, | health education, environmental - services, environmental - social, health-related community services, behaviour modification, support groups |
| Snowden et al. [268] | 2011 | general population, both genders | health promotion, primary prevention, | cognitive function, | other |
| Song et al. [269] | 2014 | 55+, both genders | health promotion, | general health, quality of life, mental health, | environmental - psychological, support groups |
| Stathokostas et al. [270] | 2012 | 65+, both genders | health promotion, | physical activity, general health, | health communication, behaviour modification |
| Stehr et al. [271] | 2012 | general population, both genders | health promotion, primary prevention, | disease oriented, physical activity, general health, | behaviour modification |
| Stern et al. [272] | 2009 | 65+, both genders | primary prevention, | disease oriented, | na |
| Stern et al. [273] | 2010 | 55+, both genders | primary prevention, | disease oriented, mental health, | na |
| Steultjens et al. [274] | 2004 | 65+, both genders | health promotion, primary prevention, | general health, quality of life, | health education, health-related community services, behaviour modification, support groups |
| Stevens et al. [275] | 2014 | other population, both genders | health promotion, | physical activity, quality of life, | health education, behaviour modification |
| Stuck et al. [276] | 2002 | 65+, both genders | primary prevention, | cognitive function, | na |
| Stuifbergen et al. [277] | 2010 | general population, both genders | health promotion, | disease oriented, general health, disability, | health education, behaviour modification |
| Sydenham et al. [278] | 2012 | 55+, both genders | primary prevention, | disease oriented, cognitive function, | na |
| Tanaka et al. [279] | 2013 | 55+, women | health promotion, | disease oriented, physical activity, | health communication, health education |
| Tanaka et al. [280] | 2012 | 55+, women | health promotion, primary prevention, | disease oriented, general health, | na |
| Tappenden et al. [281] | 2012 | 65+, both genders | health promotion, primary prevention, | disease oriented, general health, quality of life, | health education, environmental - social, environmental - psychological, health-related community services, behaviour modification |
| Teixeira et al. [282] | 2012 | 65+, both genders | health promotion, | cognitive function, | health education, behaviour modification |
| Testad et al. [283] | 2014 | 65+, both genders | primary prevention, | quality of life, cognitive function, mental health, | na |
| Theou et al. [284] | 2011 | 65+, both genders | health promotion, | physical activity, general health, quality of life, frailty, mental health, | other |
| Thomas et al. [285] | 2010 | 65+, both genders | health promotion, primary prevention, | general health, frailty, | other |
| Thomas et al. [286] | 2010 | 55+, both genders | primary prevention, | disease oriented, | na |
| Thomas et al. [287] | 2014 | 55+, both genders | primary prevention, | disease oriented, general health, mental health, | na |
| Thompson et al. [288] | 2007 | 55+, both genders | primary prevention, screening, | mental health, | na |
| Tschopp et al. [289] | 2011 | 55+, both genders | health promotion, | physical activity, | na |
| Tseng et al. [290] | 2011 | 65+, both genders | health promotion, primary prevention, | cognitive function, | environmental - social, behaviour modification |
| Turner et al. [291] | 2011 | general population, both genders | primary prevention, | disease oriented, frailty, | na |
| Udell et al. [292] | 2013 | general population, both genders | primary prevention, | disease oriented, | na |
| Vagetti et al. [293] | 2014 | 55+, both genders | health promotion, | quality of life, | other |
| Valenzuela et al. [294] | 2012 | 55+, both genders | health promotion, | physical activity, quality of life, | other |
| van der Bij et al. [295] | 2002 | other population, both genders | health promotion, | physical activity, | health communication, health education, behaviour modification |
| van Diest et al. [296] | 2013 | general population, both genders | health promotion, primary prevention, | physical activity, general health, | other |
| van Haastregt et al. [297] | 2000 | 65+, both genders | health promotion, primary prevention, social support, | general health, | health communication, health education, health-related community services |
| van Uffelen et al. [298] | 2008 | 55+, both genders | health promotion, primary prevention, | cognitive function, | na |
| Van't Leven et al. [299] | 2013 | 65+, both genders | health promotion, social support, | disease oriented, quality of life, cognitive function, | health communication, health education, environmental - services, environmental - social, environmental - psychological, health-related community services, behaviour modification, support groups |
| Vasse et al. [300] | 2010 | other population, both genders | health promotion, primary prevention, | general health, cognitive function, | health communication, environmental - social |
| Verhagen et al. [301] | 2004 | 55+, both genders | primary prevention, | frailty, | na |
| Vlaeyen et al. [302] | 2015 | 65+, both genders | primary prevention, | frailty, | na |
| Vu et al. [303] | 2002 | 65+, both genders | primary prevention, | disease oriented, | na |
| Wang et al. [304] | 2004 | general population, both genders | health promotion, primary prevention, | disease oriented, physical activity, quality of life, | health education |
| Warkentin et al. [305] | 2014 | general population, both genders | health promotion, primary prevention, | general health, quality of life, | health education, behaviour modification, |
| Watkins et al. [306] | 2014 | general population, both genders | health promotion, | general health, | health education, environmental - services, environmental - psychological, behaviour modification |
| Wayne et al. [307] | 2014 | 65+, both genders | health promotion, primary prevention, | cognitive function, | health communication, health education, behaviour modification |
| Weatherall et al. [308] | 2004 | 65+, both genders | primary prevention, | frailty, | na |
| Weatherall et al. [309] | 2004 | 65+, both genders | primary prevention, | physical activity, frailty, | na |
| Weening-Dijksterhuis et al. [310] | 2011 | 65+, both genders | health promotion, | physical activity, general health, quality of life, frailty, | health-related community services, |
| Whear et al. [311] | 2014 | 65+, both genders | health promotion, social support, | disease oriented, cognitive function, nutrition, | other |
| Whelton et al. [312] | 2002 | general population, both genders | health promotion, primary prevention, | disease oriented, general health, | other |
| Wilkins et al. [313] | 2003 | 65+, both genders | health promotion, primary prevention, social support, | disease oriented, quality of life, | health communication, health education, environmental - services, environmental - social, 1health-related community services, community mobilisation, behaviour modification, support groups, social networks |
| Willis et al. [314] | 2005 | other population, both genders | primary prevention, | disease oriented, | na |
| Wilson et al. [315] | 2003 | 55+, both genders | primary prevention, | general health, quality of life, mental health, | na |
| Windle et al. [316] | 2010 | 65+, both genders | health promotion, | general health, quality of life, mental health, | health communication, health education, community mobilisation, behaviour modification |
| Windle et al. [317] | 2007 | 65+, both genders | health promotion, | general health, mental health, | health communication, health education, environmental - social, community mobilisation, support groups |
| Winter et al. [318] | 2013 | 65+, both genders | primary prevention, | frailty, | na |
| Winterton et al. [319] | 2011 | 55+, both genders | social support, |  | na |
| Witham et al. [320] | 2010 | 55+, both genders | health promotion, primary prevention, | physical activity, quality of life, nutrition, | health communication, health education, behaviour modification, support groups |
| Woods et al. [321] | 2012 | 65+, both genders | primary prevention, | quality of life, cognitive function, | na |
| Wutzler et al. [322] | 2013 | 65+, both genders | primary prevention, | disease oriented, frailty, | na |
| Xu et al. [323] | 2014 | general population, both genders | health promotion, primary prevention, | disease oriented, | health education |
| Yamaoka et al. [324] | 2005 | other population, both genders | health promotion, primary prevention, | disease oriented, quality of life, | health education, behaviour modification |
| Yang et al. [325] | 2012 | 65+, both genders | health promotion, primary prevention, | mental health, sleep quality, | other |
| You et al. [326] | 2012 | 55+, both genders | health promotion, | general health, quality of life, frailty, mental health, | environmental - services, health-related community services, organisation culture |
| Young et al. [327] | 2011 | 65+, both genders | health promotion, | quality of life, mental health, nutrition, | health education, behaviour modification |
| Young et al. [328] | 2015 | 55+, both genders | health promotion, | cognitive function, | behaviour modification |
| Zanotto et al. [329] | 2014 | 65+, both genders | health promotion, | physical activity, general health, | health education, behaviour modification |
| Zbikowski et al. [330] | 2012 | other population, both genders | health promotion, | addiction, | health communication, health education, behaviour modification |
| Zehnacker et al. [331] | 2007 | general population, women | primary prevention, | disease oriented, physical activity, | na |
| Zheng et al. [332] | 2015 | general population, both genders | primary prevention, | disease oriented, | na |
| Zhu et al. [333] | 2013 | 55+, both genders | health promotion, | nutrition, | environmental - services, environmental - social, behaviour modification |
| Zijlstra et al. [334] | 2007 | 65+, both genders | health promotion, primary prevention, | quality of life, frailty, | health education, environmental - services, behaviour modification |

*na – not applicable

References

1. Aalbers T, Baars MAE, Olde Rikkert MGM. Characteristics of effective Internet-mediated interventions to change lifestyle in people aged 50 and older: A systematic review. Ageing Res Rev. 2011;10(4):487-497.
2. Abbott R, Whear R, Thompson-Coon J, Ukoumunne O, et al. Effectiveness of mealtime interventions on nutritional outcomes for the elderly living in residential care: A systematic review and meta-analysis. Ageing Res Rev. 2013;12(4):967-981.
3. Agmon M, Belza B, Nguyen HQ, Logsdon R, et al. A systematic review of interventions conducted in clinical or community settings to improve dual-task postural control in older adults. Clin Interv Aging. 2014;9:477-492.
4. Aguirre E, Woods RT, Spector A, Orrell M. Cognitive stimulation for dementia: A systematic review of the evidence of effectiveness from randomised controlled trials. Ageing Res Rev. 2013;12(1):253-262.
5. Anderson D, Seib C, Rasmussen L. Can physical activity prevent physical and cognitive decline in postmenopausal women? A systematic review of the literature. Maturitas. 2014;79(1):14-33.
6. Angevaren M, Aufdemkampe G, Verhaar HJJ, Aleman A, et al. Physical activity and enhanced fitness to improve cognitive function in older people without known cognitive impairment (Review). Cochrane Database Syst Rev. 2008;(3).
7. Anuruang S, Hickman LD, Jackson D, Dharmendra T, et al. Community-based interventions to promote management for older people: an integrative review. J Clin Nurs. 2014;23(15-16):2110-2120.
8. Ardern CI, Rotondi M. Knowledge Synthesis Report: The Role of Physical Activity in the Prevention and Management of Alzheimer’s Disease—Implications for Ontario. Ontario Brain Institute2013.
9. Arent SM, Landers DM, Etnier JL. The Effects of Exercise on Mood in Older Adults: A Meta-Analytic Review. J Aging Phys Act. 2000;8(4):407-430.
10. Arnold CM, Sran MM, Harrison EL. Exercise for Fall Risk Reduction in Community-Dwelling Older Adults: A Systematic Review. Physiother Can. 2008;60(4):358-72.
11. Ashworth NL, Chad KE, Harrison EL, Reeder BA, et al. Home versus center based physical activity programs in older adults (Review). Cochrane Database Syst Rev. 2005;(1).
12. Bandayrel K, Wong S. Systematic Literature Review of Randomized Control Trials Assessing the Effectiveness of Nutrition Interventions in Community-Dwelling Older Adults. J Nutr Educ Behav. 2011;43(3):251-262.
13. Barlow J, Singh D, Bayer S, Curry R, et al. A systematic review of the benefits of home telecare for frail elderly people and those with long-term conditions. J Telemed Telecare. 2007;13(4):172-179.
14. Baron RC, Melillo S, Rimer BK, Coates RJ, at al. Intervention to Increase Recommendation and Delivery of Screening for Breast, Cervical, and Colorectal Cancers by Healthcare Providers. Am J Prev Med. 2010;38(1):110 –117.
15. Batchelor F, Hill K, Mackintosh S, Said C. What Works in Falls Prevention After Stroke? A Systematic Review and Meta-Analysis. Stroke. 2010;41(8):1715-1722.
16. Bemelmans R, Gelderblom GJ, Jonker P, de Witte L a Zuyd. Socially Assistive Robots in Elderly Care: A Systematic Review into Effects and Effectiveness. J Am Med Dir Assoc. 2012;13(2):114-120.e1.
17. Berzins K, Reilly S, Abell J, Hughes J, et al. UK self-care support initiatives for older patients with long-term conditions: a review. Chronic Illn. 2009;5(1):56-72.
18. Beswick AD, Rees K, Dieppe P, Ayis S, et al. Complex interventions to improve physical function and maintain independent living in elderly people: a systematic review and meta-analysis. Lancet. 2008;371(9614):725-735.
19. Beyer W, McElhaney J, Smith D, Monto A , Nguyen-Van-Tam J, et al. Cochrane re-arranged: Support for policies to vaccinate elderlypeople against influenza. Vaccine. 2013;31(50):6030-6033.
20. Blake H, Mo P, Malik S, Thomas S. How effective are physical activity interventions for alleviating depressive symptoms in older people? A systematic review. Clin Rehabil. 2009;23(10):873-887.
21. Blankevoort CG, van Heuvelen MJG, Boersma F, Luning H, et al. Review of Effects of Physical Activity on Strength, Balance, Mobility and ADL Performance in Elderly Subjects with Dementia. Dement Geriatr Cogn Disord. 2010;30(5):392-402.
22. Bleakley CM, Charles D, Porter-Armstrong A, McNeill MDJ, et al. Gaming for Health: A Systematic Review of the Physical and Cognitive Effects of Interactive Computer Games in Older Adults. J Appl Gerontol. 2015, 34(3):NP166–NP189.
23. Blondell SJ, Hammersley-Mather R, Lennert Veerman JL. Does physical activity prevent cognitive decline and dementia?: A systematic review and meta-analysis of longitudinal studies. BMC Public Health. 2014;14:510-521.
24. Bodner ME, Dean E. Advice as a smoking cessation strategy: A systematic review and implications for physical therapists. Physiother Theory Pract. 2009;25(5–6):369–407.
25. Bolam KA, van Uffelen JGZ, Taaffe DR. The effect of physical exercise on bone density in middle-aged and older men: A systematic review. Osteoporos Int. 2013;24(11):2749-2762.
26. Bonfill Cosp X, Marzo Castillejo M, Pladevall Vila M, Marti J, et al. Strategies for increasing the participation of women in community breast cancer screening (Review). Cochrane Database Syst Rev. 2001;(1).
27. Booth V, Masud T, Connell L, Bath-Hextall F. The effectiveness of virtual reality interventions in improving balance in adults with impaired balance compared with standard or no treatment: a systematic review and meta-analysis. Clin Rehabil. 2014;28(5):419-431.
28. Bouman A, van Rossum E, Nelemans P, Kempen GIJM, et al. Effects of intensive home visiting programs for older people with poor health status: A systematic review. BMC Health Serv Res. 2008;8(1);74-84.
29. Brown CJ, Flood KL. Mobility Limitation in the Older Patient A Clinical Review. JAMA. 2013;310(11):1168-1177.
30. Brown JC, Huedo-Medina TB, Pescatello LS, Ryan SM, et al. The Efficacy of Exercise in Reducing Depressive Symptoms among Cancer Survivors: A Meta-Analysis. PLoS One. 2012;7(1).
31. Bullo V, Bergamin M, Gobbo S, Sieverdes SC, et al. The effects of Pilates exercise training on physical fitness and wellbeing in the elderly: A systematic review for future exercise prescription. Prev Med. 2005;17:1-11.
32. Cameron ID, Murray GR, Gillespie LD, Robertson MC, et al. Interventions for preventing falls in older people in nursing care facilities and hospitals (Review). Cochrane Database Syst Rev. 2010;(1).
33. Cameron ID. Gillespie LD. Robertson MC, Murray GR, et al. Interventions for preventing falls in older people in care facilities and hospitals (Review). Cochrane Database Syst Rev. 2012;(12).
34. Camilloni L, Ferroni E, Jimenez Cendales B, Pezzarossi A, et al. Methods to increase participation in organised screening programs: a systematic review. BMC Public Health. 2013;13:464.
35. Cardona-Morrell M, Rychetnik L, Morrell SL, Espinel PT, et al. Reduction of diabetes risk in routine clinical practice: are physical activity and nutrition interventions feasible and are the outcomes from reference trials replicable? A systematic review and meta-analysis. BMC Public Health. 2010;10:653.
36. Carter ND, Kannus P, Khan KM. Exercise in the Prevention of Falls in Older People. Sports Med. 2001;31(6):427-438.
37. Carvalho A, Maeve Rea I, Parimon T, Cusack BJ. Physical activity and cognitive function in individuals over 60 years of age: a systematic review. Clin Interv Aging. 2014;9:661-682.
38. Cattan M, White M, Bond J, Learmouth A. Preventing social isolation and loneliness among older people: a systematic review of health promotion interventions. Ageing Soc. 2005;25(1):41-67.
39. Chang JT, Morton SC, Rubenstein LZ, Mojica WA, et al. Interventions for the prevention of falls in older adults: systematic review and meta-analysis of randomised clinical trials. BMJ. 2004;328(7441):680.
40. Chi I, Jordan-Marsh M, Guo M, Xie B, et al. Tai chi and reduction of depressive symptoms for older adults: A meta-analysis of randomized trials. Geriatr Gerontol Int. 2013;13(1):3-12.
41. Chien Ch-L, Lee Ch-M, Wu YW, Chen T-A, et al. Home-based exercise increases exercise capacity but not quality of life in people with chronic heart failure: a systematic review. Aust J Physiother. 2008;54(2):87-93.
42. Chin AMH,. Clinical effects of reminiscence therapy in older adults: a meta-analysis of controlled trials. Hong Kong J Occup Ther. 2007;17(1):10–22.
43. Chin A Paw MJM, van Uffelen JGZ, Riphagen I, van Mechelen W.. The Functional Effects of Physical Exercise Training in Frail Older People. Sports Med. 2008;38(9):781-793.
44. Choi M, Kong S, Jung D. Computer and Internet Interventions for Loneliness and Depression in Older Adults: A Meta-Analysis. Healthc Inform Res. 2012;18(3):191-198.
45. Chou CH, Hwang CL, Wu YT. Effect of Exercise on Physical Function, Daily Living Activities, and Quality of Life in the Frail Older Adults: A Meta-Analysis. Arch Phys Med Rehabil. 2012;93(2):237-244.
46. Chou R, Dana T, Bougatsos C, Fleming C, et al. Screening Adults Aged 50 Years or Older for Hearing Loss: A Review of the Evidence for the U.S. Preventive Services Task Force. Ann Intern Med. 2011;154(5):347-355.
47. Clark IN, Taylor NF, Baker FA. Music interventions and physical activity in older adults: a systematic literature review and meta-analysis. J Rehabil Med. 2012;44(9):710-719.
48. Clegg AP, Barber SE, Young JB, Forster A, et al. Do home-based exercise interventions improve outcomes for frail older people? Findings from a systematic review. Rev Clin Gerontol. 2012;22(1):68-78.
49. Clegg A, Siddiqi N, Heaven A, Young J, et al. Interventions for preventing delirium in older people in institutional long-term care (Review). Cochrane Database Syst Rev. 2014;(1).
50. Clemson L, Mackenzie L, Ballinger C, Close JCT, et al. Environmental Interventions to Prevent Falls in Community-Dwelling Older People A Meta-Analysis of Randomized Trials. J Aging Health. 2008;20(8):954-971.
51. Cole MG, Dendukuri N. The feasibility and effectiveness of brief interventions to prevent depression in older subjects: a systematic review. Int J Geriatr Psychiatry. 2004;19(11):1019-1025.
52. Cole MG. Brief Interventions to Prevent Depression in Older Subjects: A Systematic Review of Feasibility and Effectivenes. Am J Geriatr Psychiatry. 2008;16(6):435-443.
53. Conn VS, Hafdahl AR, Mehr DR. Interventions to Increase Physical Activity Among Healthy Adults: Meta-Analysis of Outcomes. Am J Public Health. 2011;101(4):751-758.
54. Conn VS, Minor MA, Burks KJ, Rantz MJ. Integrative Review of Physical Activity Intervention Research with Aging Adults. J Am Geriatr Soc. 2003;51(8):1159-1168.
55. Conn VS, Hafdahl AR, Brown LM. Meta-analysis of quality-of-life outcomes from physical activity interventions. Nurs Res. 2009;58(3):175-183.
56. Conn VS, Valentine JC, Cooper HM. Interventions to Increase Physical Activity Among Aging Adults: A Meta-Analysis. Ann Behav Med. 2002;24(3):190-200.
57. Cooper C, Mukadam N, Katona C, Lyketsos CG, et al. Systematic review of the effectiveness of non- pharmacological interventions to improve quality of life of people with dementia. Int Psychogeriatr. 2012;24(6):856-870.
58. Costello E, Edelstein JE. Update on falls prevention for community-dwelling older adults: Review of single and multifactorial intervention programs. J Rehabil Res Dev. 2008;45(8):1135-52.
59. Crew A, Petrosky J, Byrnes K, Nelson R. The Effects of Tai Chi on Physical Functioning in Older Adults with Parkinson’s Disease. Ther Recreation J. 2015;XLIX(1):80-83.
60. Crichton GE, Bryan J, Murphy KJ, Buckley J. Review of Dairy Consumption and Cognitive Performance in Adults: Findings and Methodological Issues. Dement Geriatr Cogn Disord. 2010;30(4):352-361.
61. Crocker T, Forster A, Young J, Brown L, et al. Physical rehabilitation for older people in long-term care (Review). Cochrane Database Syst Rev. 2013;(2).
62. Crocker T, Young J, Forster A, Brown L, et al. The effect of physical rehabilitation on activities of daily living in older residents of long-term care facilities: systematic review with meta-analysis. Age Ageing. 2013;42(6):682-688.
63. Cuijpers P, Munoz RF, Clarke GN, Lewinsohn PM. Psychoeducational treatment and prevention of depression: The “coping with depression” course thirty years later. Clin Psychol Rev. 2009;29(5):449-458.
64. Cusimano MD, Kwok J, Spadafora K. Effectiveness of multifaceted fall-prevention programs for the elderly in residential care. Inj Prev. 2008;14(2):113-122.
65. Dale J, Caramlau IO, Lindenmeyer A, Williams SM. Peer support telephone calls for improving health (Review). Cochrane Database Syst Rev. 2008;(4).
66. Daniels R, van Rossum E, de Witte L, Kempen G, et al. Interventions to prevent disability in frail community-dwelling elderly: a systematic review. BMC Health Serv Res. 2008;8:278.
67. Darvishian M, Bijlsma MJ, Hak E, van den Heuvel ER. Eff ectiveness of seasonal influenza vaccine in communitydwelling elderly people: a meta-analysis of test-negative design case-control studies. Lancet Infect Dis. 2014;14(12):1228-1239.
68. Darvishiana M, Gefenaitea G, Turnerc RM, Pechlivanogloua P, Van der Hoeke W, Van den Heuvelb ER, Hak E. After adjusting for bias in meta-analysis seasonal influenza vaccine remains effective in community-dwelling elderly. J Clin Epidemiol. 2014;67(7):734-744.
69. de Kam D, Smulders E, Weerdesteyn V, Smits-Engelsman BCM. Exercise interventions to reduce fall-related fractures and their risk factors in individuals with low bone density: a systematic review of randomized controlled trials. Osteoporos Int. 2009;20(12):2111-2125.
70. de Niet G, Tiemens B, Lendemeijer B, Hutschemaekers G. Music-assisted relaxation to improve sleep quality: meta-analysis. J Adv Nurs. 2009;65(7):1356–1364.
71. de Oliveira Francisco C, de Almeida Fagundes A, Gorges B. Effects of Pilates method in elderly people: Systematic review of randomized controlled trials. J Bodyw Mov Ther. 2015;19(3):500-508.
72. de Waure C, Lauret GJ, Ricciardi W, Ferket B, et al. Lifestyle Interventions in Patients with Coronary Heart Disease A Systematic Review. Am J Prev Med. 2013;45(2):207-216.
73. DiBardino D, Cohen ER, Didwania A. Meta-analysis: multidisciplinary fall prevention strategies in the acute care inpatient population. J Hosp Med. 2012;7(6):497-503.
74. Dickens AP, Richards SH, Greaves CJ, Campbell JL. Interventions targeting social isolation in older people: a systematic review. BMC Public Health. 2011;11:647.
75. Diep L, Kwagyan J, Kurantsin-Mills J, Weir R, et al. Association of Physical Activity Level and Stroke Outcomes in Men and Women: A Meta-Analysis. J Womens Health. 2010;19(10):1815-1822.
76. Dixon L, Duncan DC, Johnson P, Kirkby L et al. Occupational therapy for patients with Parkinson’s disease (Review). Cochrane Database Syst Rev. 2007;(3).
77. Djulbegovic M, Beyth RJ, Neuberger MM, Stoffs TL, et al. Screening for prostate cancer: systematic review and metaanalysis of randomised controlled trials. BMJ. 2010;341:c4543 .
78. Dombrowski SU, Avenell A, Sniehotta FF. Behavioural Interventions for Obese Adults with Additional Risk Factors for Morbidity: Systematic Review of Effects on Behaviour, Weight and Disease Risk Factors. Obes Facts. 2010;3(6):377-396.
79. Eells K. The Use of Music and Singing to Help Manage Anxiety in Older Adults. Ment Health Pract. 2014;17(5):10-17.
80. Elkan R, Kendrick D, Dewey M, Hewitt M, et al. Effectiveness of home based support for older people: systematic review and meta­analysis. BMJ. 2001;323(7315):719-725.
81. Elkan R, Kendrick D, Hewitt M, Robinson JJA, et al. The effectiveness of domiciliary health visiting: a systematic review of international studies and a selective review of the British literature. Health Technol Assess. 2000;4(13).
82. El-Khoury F, Cassou B, Charles MA, Dargent-Molina P. The effect of fall prevention exercise programmes on fall induced injuries in community dwelling older adults: systematic review and meta-analysis of randomised controlled trials. BMJ. 2013;347:f6234.
83. Evans CJ, Goodman C, Redfern S. Maintaining independence in the cignitively intact elderly care home population: a systematic review of intervention trials. Rev Clin Gerontol. 2003;13(2): 163-174.
84. Fairhall N, Sherrington C, Clemson L, Cameron ID. Do exercise interventions designed to prevent falls affect participation in life roles? A systematic review and meta-analysis. Age Ageing. 2011;40(6):666-674.
85. Faucounau V, Wu Y-H, Boulay M, De Rotrou J, et al. Cognitive intervention programmes on patients affected by mild cognitive impairment: a promosing intervention tool for MCI?. J Nutr Health Aging. 2010;14(10):31-35.
86. Felix HC, West DS. Effectiveness of Weight Loss Interventions for Obese Older Adults. Am J Health Promot. 2013;27(3):191-199.
87. Ferreira ML, Sherrington C, Smith K, Carswell P, et al. Physical activity improves strength, balance and endurance in adults aged 40–65 years: a systematic review. J Physiother. 2012;58(3):145-156.
88. Ferroni E, Camilloni L, Jimenez B, Furnari G, et al. How to increase uptake in oncologic screening: a systematic review of studies comparing population-based screening programs and spontaneous access. Prev Med. 2012;55(6):587-596.
89. Forbes D, Forbes SC, Blake CM, Thiessen EJ, et al. Exercise programs for people with dementia (Review). Cochrane Database Syst Rev. 2015;(4).
90. Forsman AK, Nordmyr J, Wahlbeck K. Psychosocial interventions for the promotion of mental health and the prevention of depression among older adults. Health Promot Int. 2011;26(S1).
91. Forsman AK, Schierenbeck I, Wahlbeck K. Psychosocial Interventions for the Prevention of Depression in Older Adults: Systematic Review and Meta-Analysis. J Aging Health. 2011;23(3):387–416.
92. Gagliardi AMZ, Gomes Silva BN, Torloni MR, Soares BGO. Vaccines for preventing herpes zoster in older adults (Review). Cochrane Database Syst Rev. 2012;(10).
93. Gard T, Hölzel BK, Lazar SW. The potential effects of meditation on age-related cognitive decline: a systematic review. Ann N Y Acad Sci. .
94. Gardner MP, Adams A, Jeffreys M. Interventions to Increase the Uptake of Mammography amongst Low Income Women: A Systematic Review and Meta-Analysis. PLoS One. 2013;8(2).
95. Gates N, Fiatarone Singh MA, Sachdev PS, Valenzuela M. The Effect of Exercise Training on Cognitive Function in Older Adults with Mild Cognitive Impairment: A Meta-analysis of Randomized Controlled Trials. Am J Geriatr Psychiatry. 2013;21(11):1086-1097.
96. Gellis ZD, Kang-Yi C. Meta-Analysis of the Effect of Cardiac Rehabilitation Interventions on Depression Outcomes in Adults 64 Years of Age and Older. Am J Cardiol. 2012;110(9):1219-1224.
97. Geraedts H, Zijlstra A, Bulstra SK, Stevens M, et al. Effects of remote feedback in home-based physical activity interventions for older adults: A systematic review. Patient Educ Couns. 2013;91(1):14-24.
98. Gillespie LD, Gillespie WJ, Robertson MC, Lamb SE, et al. Interventions for preventing falls in elderly people (Review). Cochrane Database Syst Rev. 2003;(4).
99. Gillespie LD, Robertson MC, Gillespie WJ, Lamb SE, et al. Interventions for preventing falls in older people living in the community (Review). Cochrane Database Syst Rev. 2009;(2).
100. Gillespie LD, Robertson MC, Gillespie WJ, Sherrington C, et al. Interventions for preventing falls in older people living in the community (Review). Cochrane Database Syst Rev. 2013;(1).
101. Gine-Garriga M, Roque-Figuls M, Coll-Planas L, Sitja-Rabert M, et al. Physical Exercise Interventions for Improving Performance-Based Measures of Physical Function in Community-Dwelling, Frail Older Adults: A Systematic Review and Meta-Analysis. Arch Phys Med Rehabil. 2014;95(4):753-769.
102. Gleeson M, Sherrington C, Keay L. Exercise and physical training improve physical function in older adults with visual impairments but their effect on falls is unclear: a systematic review. J Physiother. 2014;60(3):130-135.
103. Gnoato Zotz TG, Cunha Loureiro AP, Valderramas SR, Silveira Gomes AR. Stretching—An Important Strategy to Prevent Musculoskeletal Aging. Top Geriatr Rehabil. 2014;30(4):246-255.
104. Gobbo S, Bergamin M, Sieverdes JC, Ermolao A, et al. Effects of exercise on dual-task ability and balance in older adults: A systematic review. Arch Gerontol Geriatr. 2014;58(2):177-187.
105. Goodwin V, Jones-Hughes T, Thompson-Coon J, Boddy K, et al. Implementing the evidence for preventing falls among community-dwelling older people: A systematic review. J Safety Res. 2011;42(6):443–451.
106. Goodwin VA, Abbott RA, Whear R, Bethel A, et al. Multiple component interventions for preventing falls and fall-related injuries among older people: systematic review and meta-analysis. BMC Geriatr. 2014;14:15.
107. Gotzsche PC, Jorgensen KJ. Screening for breast cancer with mammography (Review). Cochrane Database Syst Rev. 2013;(6).
108. Gould RL, Coulson MC, Patel N, Highton-Williamson E, et al. Interventions for reducing benzodiazepine use in older people: meta-analysis of randomised controlled trials. Br J Psychiatry. 2014;204(2):98-107.
109. Gregory H, Watson MC. The effectiveness of Tai Chi as a fall prevention intervention for older adults: a systematic review. Int J Health Promot Educ. 2009;47(3):94-100.
110. Gu MO, Conn VS. Meta-Analysis of the Effects of Exercise Interventions on Functional Status in Older Adults. Res Nurs Health. 2008;31(6):594-603.
111. Guo J-L, Tsai Y-Y, Liao J-Y, Tu H-M, et al. Interventions to reduce the number of falls among older adults with/without cognitive impairment: an exploratory meta-analysis. Int J Geriatr Psychiatry. 2014;29(7):661-669.
112. Hackshaw A. EUSOMA review of mammography screening. Ann Oncol. 2003;14(8):1193-1195.
113. Hamilton CJ, Swan VJD, Jamal SA. The effects of exercise and physical activity participation on bone mass and geometry in postmenopausal women: a systematic review of pQCT studies. Osteoporos Int. 2010;21(1):11-23.
114. Harling A, Simpson JP. A systematic review to determine the effectiveness of Tai Chi in reducing falls and fear of falling in older adults. Phys Ther Rev. 2008;13(4):237-248.
115. Harris R, Yeatts J, Kinsinger L. Breast cancer screening for women ages 50 to 69 years a systematic review of observational evidence. Prev Med. 2011;53(3):108-114.
116. Hauer K, Becker C, Lindemann U, Beyer N. Effectiveness of Physical Training on Motor Performance and Fall Prevention in Cognitively Impaired Older Persons. Am J Phys Med Rehabil. 2006;85(10):847-857.
117. Hayes JH, Barry MJ. Screening for Prostate Cancer With the Prostate-Specific Antigen Test. A Review of Current Evidence. JAMA. 2014;311(11):1143-1149.
118. Healey F, Oliver D, Milne A, Connelly JB.. The effect of bedrails on falls and injury: a systematic review of clinical studies. Age Ageing. 2008;37(4):368-378.
119. Heaven B, Brown LJE, White M, Errington L, et al. Supporting Well-Being in Retirement through Meaningful Social Roles: Systematic Review of Intervention Studies. Milbank Q. 2013;91(2):222-287.
120. Heyn PC, Johnson KE, Kramer AF. Endurance and Strength Training Outcomes on Cognitively Impaired and Cognitively Intact Older Adults: A Meta-analysis. J Nutr Health Aging. 2008;12(6):401-409.
121. Hill-Westmoreland EE, Soeken K, Spellbring AM. A Meta-Analysis of Fall Prevention Programs for the Elderly. How Effective Are They?. Nurs Res. 2002;51(1):1-8.
122. Hobbs N, Godfrey A, Lara J, Errington L, et al. Are behavioral interventions effective in increasing physical activity at 12 to 36 months in adults aged 55 to 70 years? A systematic review and meta-analysis. BMC Med. 2013;11:75.
123. Howe TE, Rochester L, Neil F, Skelton DA, et al. Exercise for improving balance in older people (Review). Cochrane Database Syst Rev. 2011;(11).
124. Huang G, Gibson CA, Tran ZV, Osness WH. Controlled Endurance Exercise Training and VO2max Changes in Older Adults: A Meta-Analysis. Prev Cardiol. 2005;8(4):217-225.
125. Huang G, Shi X, Gibson CA, Huang SC, et al. Controlled aerobic exercise training reduces resting blood pressure in sedentary older adults. Blood Press. 2013;22(6):386-394.
126. Humphrey L, Helfand M, Chan BKS, Wolf SH. Breast Cancer Screening: A Summary of the Evidence for the U.S. Preventive Services Task Force. Ann Intern Med. 2002;137(5 Part 1):347-60.
127. Huss A, Stuck AE, Rubenstein LZ, Egger M, et al. Multidimensional Preventive Home Visit Programs for Community-Dwelling Older Adults: A Systematic Review and Meta-Analysis of Randomized Controlled Trials. J Gerontol A Biol Sci Med Sci. 2008;63A(3):298-307.
128. Ilic D, O’Connor D, Green S, Wilt TJ. Screening for prostate cancer: an updated Cochrane systematic review. BJU Int. 2011;107(6):882-891.
129. Ilic D, Neuberger MM, Djulbegovic M, Dahm P. Screening for prostate cancer (Review). Cochrane Database Syst Rev. 2013;(1).
130. Ishigaki EY, Ramos LG, Carvalho ES, Lunardi AC. Effectiveness of muscle strengthening and description of protocols for preventing falls in the elderly: a systematic review. Braz J Phys Ther. 2014;18(2):111-118.
131. Jefferson T, Rivetti D, Rivetti A, Rudin M, et al. Efficacy and effectiveness of influenza vaccines in elderly people: a systematic review. Lancet. 2005;366(9492):1165-1174.
132. Jefferson T, Di Pietrantonj C, Al-Ansary LA, Ferroni E, et al. Vaccines for preventing influenza in the elderly (Review). Cochrane Database Syst Rev. 2010;(2).
133. Joe J, Demiris G. Older adults and mobile phones for health: A review. J Biomed Inform. 2013;46(5):947-954.
134. Jonker AAGC, Comijs HC, Knipscheer KCPM, Deeg DJH. Promotion of self-management in vulnerable older people: a narrative literature review of outcomes of the Chronic Disease Self-Management Program (CDSMP). Eur J Ageing. 2009; 6(4):303–314.
135. Kachouie R, Sedighadeli S, Khosla R, Chu MT. Socially Assistive Robots in Elderly Care: A Mixed-Method Systematic Literature Review. Int J Hum Comput Interact. 2014;30(5):369-393.
136. Kelley GA, Kelley KS. Aerobic Exercise and Resting Blood Pressure in Older Adults: A Meta-analytic Review of Randomized Controlled Trials. J Gerontol A Biol Sci Med Sci. 2001;56(5):M298-303.
137. Kelley GA, Kelley KS, Tran ZV. Exercise, Lipids, and Lipoproteins in Older Adults: A Meta-Analysis.. Prev Cardiol. 2005 ;8(4):206–214.
138. Kelley GA, Kelley KS, Kohrt WM. Exercise and bone mineral density in men: A meta-analysis of randomized controlled trials. Bone. 2013;53(1):103-111.
139. Kelley GA, Kelley KS, Tran ZV. Exercise and Lumbar Spine Bone Mineral Density in Postmenopausal Women: A Meta-Analysis of Individual Patient Data. J Gerontol A Biol Sci Med Sci. 2002;57(9):M599-M604.
140. Kelly ME, Loughrey D, Lawlor BA, Robertson IH, et al. impact of exercise on the cognitive functioning of healthy older adults: A systematic review and meta-analysis. Ageing Res Rev. 2014;16:12-31.
141. Kelly ME, Loughrey D, Lawlor BA, Robertson IH, et al. The impact of cognitive training and mental stimulation on cognitive and everyday functioning of healthy older adults: A systematic review and meta-analysis. Ageing Res Rev. 2014;15(1):28-43.
142. Kelly GA, Kelley KS, Tran ZV. Resistance Training and Bone Mineral Density in Women A Meta-Analysis of Controlled Trials. Am J Phys Med Rehabil. 2001;80(1):65-77.
143. Kemmler W, Häberle L, von Stengel S. Effects of exercise on fracture reduction in older adults A systematic review and meta-analysis. Osteoporos Int. 2013;24(7):1937-50.
144. Kendrick D, Kumar A, Carpenter H, Zijlstra GAR, et al. Exercise for reducing fear of falling in older people living in the community (Review). Cochrane Database Syst Rev. 2014;(11).
145. Kosse MN, Brands K, Bauer JM, Hortobagyi T, et al. Sensor technologies aiming at fall prevention in institutionalized old adults: A synthesis of current knowledge. Int J Med Inform. 2013;82(9):743-752.
146. Kösters JP, Gotzsche PC. Regular self-examination or clinical examination for early detection of breast cancer (Review). Cochrane Database Syst Rev. 2003;(2).
147. Kotwal S, Jun M, Sullivan D, Perkovic V, et al. Omega 3 Fatty Acids and Cardiovascular Outcomes Systematic Review and Meta-Analysis. Circ Cardiovasc Qual Outcomes. 2012;5(6):808-818.
148. Kua A, Korner-Bitensky N, Desrosieres J, Man-Son-Hing M, et al. Older driver retraining: A systematic review of evidence of effectiveness. J Safety Res. 2007;38(1):81-90.
149. Kueider AM, Parisi JM, Gross AL, Rebok GW. Computerized Cognitive Training with Older Adults: A Systematic Review. PLoS One. 2012;7(7).
150. Lai P, Chua SS, Chan SP. A systematic review of interventions by healthcare professionals on community-dwelling postmenopausal women with osteoporosis. Osteoporos Int. 2010:21(10):1637-1656.
151. Lara J, Hobbs N, Moynihan PJ, Meyer TD, et al. Effectiveness of dietary interventions among adults of retirement age: a systematic review and meta-analysis of randomized controlled trials. BMC Med. 2014;12:60.
152. Lau D, Hu J, Majumdar SR, Storie DA, et al. Interventions to Improve Influenza and Pneumococcal Vaccination Rates Among Community-Dwelling Adults: A Systematic Review and Meta-Analysis. Ann Fam Med. 2012;10(6):538-46.
153. Law LLF, Barnett F, Yau MK, Gray MA. Effects of combined cognitive and exercise interventions on cognition in older adults with and without cognitive impairment: A systematic review. Ageing Res Rev. 2014;15(1):61-75.
154. Lee MS, Lee EN, Kim JI, Ernst E. Tai chi for lowering resting blood pressure in the elderly: a systematic review. J Eval Clin Pract. 2010;16(4):818-824.
155. Lee MS, Pittler MH, Shin B-C, Ernst E. Tai chi for osteoporosis: a systematic review. Osteoporos Int. 2008;19(2):139-146.
156. Lee SY, Franchetti MK, Imanbayev A, Gallo JJ, et al. Non-pharmacological prevention of major depression among community-dwelling older adults: A systematic review of the efficacy of psychotherapy interventions. Arch Gerontol Geriatr. 2012;55(3):522-529.
157. Legg L, Drummond A, Langhorne P. Occupational therapy for patients with problems in activities of daily living after stroke (Review). Cochrane Database Syst Rev. 2006;(4).
158. Legler J, Meissner HI, Coyne C, Breen N. The Effectiveness of Interventions To Promote Mammography among Women with Historically Lower Rates of Screening. Cancer Epidemiol Biomarkers Prev. 2002;11(1):59-71.
159. Leung DPK, Chan CKL, Tsang HWH, Tsang WWN, el al. Tai Chi as an Intervention to Improve Balance and Reduce Falls in Older Adults: A Systematic and Meta-analytical review. Altern Ther Health Med. 2011;17(1):40-48.
160. Leung GM, Lam T-H, Thach TQ, Hedley AJ. Will Screening Mammography in the East Do More Harm than Good?. Am J Public Health. 2002;92(11):1841-1846.
161. Liebel DV, Friedman B, Watson NM, Powers BA. Review of Nurse Home Visiting Interventions for Community-Dwelling Older PersonsWith Existing Disability. Med Care Res Rev. 2009;66(2):119-146.
162. Littbrand H, Stenvall M, Rosendahl E. Applicability and effects of physical exercise on physical and cognitive functions and activities of daily living among people with dementia: a systematic review.. Am J Phys Med Rehabil. 2011;90(6):495-518.
163. Liu W, Cheon J, Thomas SA. Interventions on mealtime difficulties in older adults with dementia: A systematic review. Int J Nurs Stud. 2014;51(1):14-27.
164. Liu S, Dunford SD, Leung YW, Brooks D, et al. Reducing Blood Pressure With Internet-Based Interventions: A Meta-analysis. Can J Cardiol. 2013;29(5):613-621.
165. Liu CJ, Latham NK.. Progressive resistance strength training for improving physical function in older adults (Review). Cochrane Database Syst Rev. 2009;(3).
166. Liu Y, Hu F, Li D, Wang F, et al. Does Physical Activity Reduce the Risk of Prostate Cancer? A Systematic Review and Meta-analysis. Eur Urol. 2011;60(5):1029-1044.
167. Lock CA, Lecouturier J, Mason JM, Dickinson HO. Lifestyle interventions to prevent osteoporotic fractures: a systematic review. Osteoporos Int. 2006;17(1):20–28.
168. Logghe IHJ, Verhagen AP, Rademaker ACHJ, Bierma-Zeinstra SMA et al. The effects of Tai Chi on fall prevention, fear of falling and balance in older people: A meta-analysis. Prev Med. 2010;51(3-4):222-227.
169. Lopopolo RB, Greco M, Sullivan D, Crail RL, et al. Effect of Therapeutic Exercise on Gait Speed in Community-Dwelling Elderly People: A Meta-analysis. Phys Ther. 2006;86(4):520-540.
170. Low S, Ang LW, Goh KS, Chew SK. A systematic review of the effectiveness of Tai Chi on fall reduction among the elderly. Arch Gerontol Geriatr. 2009;48(3):325-331.
171. Luna EJA, Gattas VL. Effectiveness of the Brazilian influenza vaccination policy, a systematic review. Rev Inst Med Trop Sao Paulo. 2010;52(4):175-181.
172. Manafo E, Wong S. Health literacy programs for older adults: a systematic literature review. Health Educ Res. 2012;27(6):947-960.
173. Mandelblatt J, Saha S, Teutsch S, Hoerger T, et al. The Cost-Effectiveness of Screening Mammography beyond Age 65 Years: A Systematic Review for the U.S. Preventive Services Task Force. Ann Intern Med. 2003;139(10):835-842 .
174. Manzoli L, Ioannidis JPA, Flacco ME, De Vito C, et al. Effectiveness and harms of seasonal and pandemic influenza vaccines in children, adults and elderly. Hum Vaccin Immunother. 2012;8(7):851-862.
175. March S, Torres E, Ramos M, Ripoll J, et al. Adult community health-promoting interventions in primary health care: A systematic review. Prev Med. 2015;76 Suppl:S94-104.
176. Markle-Reid M, Browne G, Weir R, Gafni A, et al. The Effectiveness and Efficiency of Home-Based Nursing Health Promotion for Older People: A Review of the Literature. Med Care Res Rev. 2006;63(5):531-569.
177. Marks R, Allegrante JP. Falls-prevention programs for older ambulatory community dwellers: from public health research to health promotion policy. Int J Public Health. 2004;49(3):171-178.
178. Marmot M, et al. The benefits and harms of breast cancer screening: an independent review. Lancet. 2012;380(9855):1778-1786 .
179. Marques EA, Mota J, Carvalho J. Exercise effects on bone mineral density in older adults: meta-analysis of randomized controlled trials. Age. 2012;34(6):1493-1515.
180. Marshall S, Bauer J, Capra S, IsenrIng E. Are informal carers and community care workers effective in managing malnutrition in the older adult community? A systematic review of current evidence. J Nutr Health Aging. 2013;17(8):645-651.
181. Martin M, Clare L, Altgassen AM, Cameron MH, et al. Cognition-based interventions for healthy older people and people with mild cognitive impairment (Review). Cochrane Database Syst Rev. 2011;(1).
182. Martin JT, Wolf A, Moore JL, Rolenz E, et al. The Effectiveness of Physical Therapist– Administered Group-Based Exercise on Fall Prevention: A Systematic Review of Randomized Controlled Trials. J Geriatr Phys Ther. 2013;36(4):182-193.
183. Martinez F, Tobar C, Hill N. Preventing delirium: should non-pharmacological, multicomponent interventions be used? A systematic review and meta-analysis of the literature. Age Ageing. 2015;44(2):196-204.
184. Martyn-St James M, Carroll S. Meta-analysis of walking for preservation of bone mineral density in postmenopausal women. Bone. 2008;43(3):521-531.
185. Massat NJ, Moss SM, Halloran SP, Duffy SW. Screening and Primary prevention of Colorectal Cancer: a Review of sex-specific and site-specific differences. J Med Screen. 2013;20(3):125-148.
186. Maujean A, Pepping CA, Kendall E. A Systematic Review of Randomized Controlled Studies of Art Therapy. Art Ther J Am Art Ther Assoc. 2014;31(1):37-44.
187. McClure RJ, Turner C, Peel N, Spinks A, et al. Population-based interventions for the prevention of fall-related injuries in older people (Review). Cochrane Database Syst Rev. 2005;(1).
188. McDaid D, Park A. Investing in mental health and well-being: findings from the DataPrev project. Health Promot Int. 2011;26(1).
189. McDermott MS, While AE. Maximizing the healthcare environment: A systematic review exploring the potential of computer technology to promote self-management of chronic illness in healthcare settings. Patient Educ Couns. 2013;92(1):13-22.
190. McGrath C, Zhang W, Lo EC. A review of the effectiveness of oral health promotion activities among elderly people. Gerodontology. 2009;26(2):85-96.
191. McNamara RJ, McKeough ZJ, McKenzie DK, Alison JA. Water-based exercise training for chronic obstructive pulmonary disease (Review). Cochrane Database Syst Rev. 2013;(12).
192. McPhate L, Simek EM, Haines TP. Program-related factors are associated with adherence to group exercise interventions for the prevention of falls: a systematic review. J Physiother. 2013;59(2):81-92.
193. Michael YL, Whitlock EP, Lin JS, Fu R, et al. Primary Care–Relevant Interventions to Prevent Falling in Older Adults: A Systematic Evidence Review for the U.S. Preventive Services Task Force. Ann Intern Med. 2010;153(12):815-825.
194. Michiels B, Govaerts F, Remmena R, Vermeirea E, et al. A systematic review of the evidence on the effectiveness and risks of inactivated influenza vaccines in different target group. Vaccine. 2011;29(49):9159-9170.
195. Michikawa T, Nishiwaki Y, Takebayashi T, Toyama Y. One-leg standing test for elderly populations. J Orthop Sci. 2009;14(5):675-685.
196. Miller KJ, Adair BS, Pearce AJ, Said CM, et al. Effectiveness and feasibility of virtual reality and gaming system use at home by older adults for enabling physical activity to improve health-related domains: a systematic review. Age Ageing. 2014;43(2):188-195.
197. Miller CT, Fraser SF, Levinger I, Straznicky NE, et al. The Effects of Exercise Training in Addition to Energy Restriction on Functional Capacities and Body Composition in Obese Adults during Weight Loss: A Systematic Review. PLoS One. 2013;8(11):e81692.
198. Moayyeri A. The Association Between Physical Activity and Osteoporotic Fractures: A Review of the Evidence and Implications for Future Research. Ann Epidemiol. 2008;18(11):827-835.
199. Moberley S, Holden J, Tatham DP, Andrews RM. Vaccines for preventing pneumococcal infection in adults. Cochrane Database Syst Rev. 2013;(1).
200. Montgomery P, Dennis J. A systematic review of non-pharmacological therapies for sleep problems in later life. Sleep Med Rev. 2004;8(1):47-62.
201. Montgomery P, Dennis JA. Cognitive behavioural interventions for sleep problems in adults aged 60+ (Review). Cochrane Database Syst Rev. 2003;(1).
202. Moran PS, Flattery MJ, Teljeur C, Ryan M, et al. Effectiveness of systematic screening for the detection of atrial fibrillation (Review). Cochrane Database Syst Rev. 2013;(4).
203. Moreland J, Richardson J, Chan DH, O'Neill J, et al. Evidence-based guidelines for the secondary prevention of falls in older adults.. Gerontology. 2003;49(2):93-116.
204. Morris ME, Adair B, Ozzane E, Kurowski W, et al. Smart technologies to enhance social connectedness in older people who live at home.. Australas J Ageing. 2014;33(3):142-152.
205. Müller-Riemenschneider F, Reinhold T, Nocon M, Willich SN. Long-term effectiveness of interventions promoting physical activity: A systematic review. Prev Med. 2008;47(4):354-368.
206. Mulligan NF, Tschoepe BA, Smith MB. Balance Retraining in Community-Dwelling Older Adults. Top Geriatr Rehabil. 2014;30(2):117-126.
207. Murphy M, Nevill A, Murtagh E, Holder R. The effect of walking on fitness, fatness and resting blood pressure: A meta-analysis of randomised, controlled trials. Prev Med. 2007;44(5):377-385.
208. Naqvi R, Liberman D, Rosenberg J, Alston J, et al. Preventing cognitive decline in healthy older adults. Can Med Assoc J. 2013;185(10) :881-885.
209. Ndiaye SM, Hopkins DP, Shefer AM, Hinman AR, et al. Interventions to Improve Influenza, Pneumococcal Polysaccharide, and Hepatitis B Vaccination Coverage Among High-Risk Adults. Am J Prev Med. 2005;28(5S):248-279.
210. Neidrick TJ, Fick DM, Loeb SJ. Physical activity promotion in primary care targeting the older adult. J Am Acad Nurse Pract. 2012;24(7):405-416.
211. Nelson HD, Tyne K, Naik A, Bougatsos C, et al. Screening for Breast Cancer: Systematic Evidence Review Update for the U. S. Preventive Services Task Force. Ann Intern Med. 2009;151(10):727–742.
212. Netz Y, Wu MJ. Physical Activity and Psychological Well-Being in Advanced Age: A Meta-Analysis of Intervention Studies. Psychol Aging. 2005;20(2):272-284.
213. Neyens JC, van Haastregt JC, Dijcks BP, Martens M, et al. Effectiveness and Implementation Aspects of Interventions for Preventing Falls in Elderly People in Long-Term Care Facilities: A Systematic Review of RCTs. J Am Med Dir Assoc. 2011;12(6):410-425.
214. Nichol KL. Influenza Vaccination in the Elderly Impact on Hospitalisation and Mortality. Drugs Aging. 2005;22(6):495-515.
215. Nickson C, Mason KE, English DR, Kavanagh AM. Mammographic Screening and Breast Cancer Mortality: A Case–Control Study and Meta-analysis. Cancer Epidemiol Biomarkers Prev. 2012;21(9):1479-1488.
216. Nikander R, Sievänen H, Heinonen A, Daly RM. Targeted exercise against osteoporosis: A systematic review and meta-analysis for optimising bone strength throughout life. BMC Med. 2010;8:47.
217. O`Conor EA, Whitlock EP, Gaynes B, Beil TL. Screening for Depression in Adults and Older Adults in Primary Care: An Updated Systematic Review. U.S. Preventive Services Task Force Evidence Syntheses2009;Report No.:10-05143-EF-1.
218. O’Hare MP, Pryde SJ, Gracey JH. A systematic review of the evidence for the provision of walking frames for older people. Phys Ther Rev. 2013;18(1):11-23.
219. Öhman H, Savikko N, Strandberg TE, Pitkälä KH. Effect of Physical Exercise on Cognitive Performance in Older Adults with Mild Cognitive Impairment or Dementia: A Systematic Review. Dement Geriatr Cogn Disord. 2014;38(5-6):347-365.
220. Oliver D, Connelly JB, Victor CR, Shaw FE, et al. Strategies to prevent falls and fractures in hospitals and care homes and effect of cognitive impairment: systematic review and meta-analyses. BMJ. 2007;334(7564):82.
221. Ooi CP, Loke SC, Yassin Z, Hamid TA. Carbohydrates for improving the cognitive performance of independent-living older adults with normal cognition or mild cognitive impairment (Review). Cochrane Database Syst Rev. 2011;(4).
222. Orr R, Raymond J, Singh MF. Efficacy of Progressive Resistance Training on Balance Performance in Older Adults. Sports Med. 2008;38(4):317-343.
223. Oyama H, Sakashita T, Ono Y, Goto M, et al. Effect of Community-based Intervention Using Depression Screening on Elderly Suicide Risk: A Meta-analysis of the Evidence from Japan. Community Ment Health J. 2008;44(5):311-320.
224. Paap E, Verbeek ALM, Puliti D, Paci E, Broeders MJM. Breast cancer screening case–control study design: impact on breast cancer mortality. Ann Oncol. 2010;22(4):863-869.
225. Pace LE, Keating NL. A Systematic Assessment of Benefits and Risks to Guide Breast Cancer Screening Decisions. JAMA. 2014;311(13):1327-1335.
226. Park SH, Han KS, Kang CB. Effects of exercise programs on depressive symptoms, quality of life, and self-esteem in older people: A systematic review of randomized controlled trials.. Appl Nurs Res. 2014;27(4):219-26.
227. Parker MJ, Gillespie WJ, Gillespie LD. Effectiveness of hip protectors for preventing hip fractures in elderly people: systematic review. BMJ. 2006;332(7541):571-574.
228. Parker SG, Lee SD, Fadayevatan R. Co-ordinating discharge of elderly people from hospital to the community. Evid Base Healthc Publ Health. 2004;8(6):332–334.
229. Passos GS, Rollemberg Poyares DL, Goncalves Santana M, Tufik S, et al. Is exercise an alternative treatment for chronic insomnia?. Clinics (Sao Paulo). 2012;67(6):653-660.
230. Patel NK, Newstead AH, Ferrer RL. The Effects of Yoga on Physical Functioning and Health Related Quality of Life in Older Adults: A Systematic Review and Meta-Analysis. J Altern Complement Med. 2012;18(10):902-917.
231. Perry M, Draskovic I, Lucassen P, Vernooij-Dassen M, et al. Effects of educational interventions on primary dementia care: A systematic review. Int J Geriatr Psychiatry. 2010;26(1):1-11.
232. Peterson MD, Rhea MR, Sen A, Gordon PM. Resistance exercise for muscular strength in older adults: A meta-analysis. Ageing Res Rev. 2010;9(3):226-237.
233. Peterson MD, Sen A, Gordon PM. Influence of Resistance Exercise on Lean Body Mass in Aging Adults: A Meta-Analysis. Med Sci Sports Exerc. 2011;43(2):249-258.
234. Petridou ET, Manti EG, Ntinapogias AG, Negri E, et al. What Works Better for Community-Dwelling Older People at Risk to Fall? A Meta-Analysis of Multifactorial Versus Physical Exercise-Alone Interventions. J Aging Health. 2009;21(5):713-729.
235. Pitkälä K, Savikko N, Poysti M, Strandberg T, et al. Efficacy of physical exercise intervention on mobility and physical functioning in older people with dementia: A systematic review. Exp Gerontol. 2013;48(1):85-93.
236. Plawecki K, Chapman-Novakofski K. Bone Health Nutrition Issues in Aging. Nutrients2010;2(11):1086-1105.
237. Ploeg J, Feightner J, Hutchison B, Patterson C, et al. Eff ectiveness of preventive primary care outreach interventions aimed at older people. Can Fam Physician. 2005;51:1244-1245..
238. Polisena J, Tran K, Cimon K, Hutton B, et al. Home telehealth for chronic obstructive pulmonary disease: a systematic review and meta-analysis. J Telemed Telecare. 2010;16(3):120-127.
239. Potter R, Ellard D, Rees K, Thorogood M. A systematic review of the effects of physical activity on physical functioning, quality of life and depression in older people with dementia. Int J Geriatr Psychiatry. 2011;26(10):1000-1011.
240. Primack BA, Carroll MV, McNamara M, Klem ML, et al. Role of Video Games in Improving Health-Related Outcomes: A Systematic Review. Am J Prev Med. 2012;42(6):630-638.
241. Rand D, Miller WC, Yiu J, Eng JJ. Interventions for addressing low balance confidence in older adults: a systematic review and meta-analysis. Age Ageing. 2011;40(3):297-306.
242. Raymond MJ, Bramley-Tzerefos RE, Jeffs KJ, Winter A, et al. Systematic Review of High-Intensity Progressive Resistance Strength Training of the Lower Limb Compared With Other Intensities of Strength Training in Older Adults. Arch Phys Med Rehabil. 2013;94(8):1458-1472.
243. Reeder B, Meyer E, Lazar A, Chaudhuri S, et al. Framing the evidence for health smart homes and home-based consumer health technologies as a public health intervention for independent aging: a systematic review. Int J Med Inform. 2013;82(7):565-579.
244. Reijnders J, van Heugten C, van Boxtel M. Cognitive interventions in healthy older adults and people with mild cognitive impairment: A systematic review. Ageing Res Rev. 2013;12(1):263-275.
245. Roberts H, Walker-Dilks C, Sivjee K, Ung Y, et al. Screening High-Risk Populations for Lung Cancer. J Thorac Oncol. 2013;8(10):1232-1237.
246. Robertson MC, Campbell AJ, Gardner MM, Devlin N. Preventing Injuries in Older People by Preventing Falls: A Meta-Analysis of Individual-Level Data. J Am Geriatr Soc. 2002;50(5):905-11.
247. Rodrigues EV, Valderramas SR, Rossetin LL, Silveira Gomes AR. Effects of Video Game Training on the Musculoskeletal Function of Older Adults. Top Geriatr Rehabil. 2014;30(4):238-245.
248. Roets-Merken LM, Draskovic I, Zuidema SU, van Erp WS, et al. Effectiveness of rehabilitation interventions in improving emotional and functional status in hearing or visually impaired older adults: a systematic review with meta-analyses. Clin Rehabil. 2015;29(2):107–119.
249. Rogers C, Larkey LK, Keller C. A Review of Clinical Trials of Tai Chi and Qigong in Older Adults. West J Nurs Res. 2009;31(2):245–279.
250. Ross LA, Schmidt EL, Ball K. Interventions to maintain mobility: What works?. Accid Anal Prev. 2013;61:167-196.
251. Rubenstein LZ. Falls in older people: epidemiology, risk factors and strategies for prevention. Age Ageing. 2006;35(S2):ii37-ii41.
252. Rydwik E, Frandin K, Akner G. Effects of physical training on physical performance in institutionalised elderly patients (70+) with multiple diagnoses. Age Ageing. 2004;33(1):13-23.
253. Ryhanen AM, Siekkinen M, Rankinen S, Korvenranta H, Leino-Kilpi H. The effects of Internet or interactive computer-based patient education in the field of breast cancer: A systematic literature review. Patient Educ Couns. 2010;79(1):5-13.
254. Santesso N, Carrasco-Labra A, Brignardello-Petersen R. Hip protectors for preventing hip fractures in older people (Review). Cochrane Database Syst Rev. 2013;(3).
255. Sawka AM, Boulos P, Beattie K, Papaioannou A, et al. Hip protectors decrease hip fracture risk in elderly nursing home residents: a Bayesian meta-analysis. J Clin Epidemiol. 2007;60(4):336-344.
256. Sawka AM, Boulos P, Beattie K, Thabane L, et al. Do hip protectors decrease the risk of hip fracture in institutional and community-dwelling elderly? A systematic review and meta-analysis of randomized controlled trials. Osteoporos Int. 2005;16(12):1461–1474.
257. Schoene D, Valenzuela T, Lord SR, de Bruin ED. The effect of interactive cognitive-motor training in reducing fall risk in older people: a systematic review. BMC Geriatr. 2014;14:107.
258. Schroeder K, Fahey T, Ebrahim S This. Interventions for improving adherence to treatment in patients with high blood pressure in ambulatory settings (Review). Cochrane Database Syst Rev. 2004;(3).
259. Schwenk M, DeHaven Jordan E, Honarvararaghi B, Mohler J, et al. Effectivenes of Foot and Ankle Exercise Programs on Reducing the Risk of Falling in Older Adults. J Am Med Dir Assoc. 2013;103(6):534-547.
260. Sharp K, Hewitt J. Dance as an intervention for people with Parkinson’s disease: A systematic review and meta-analysis. Neurosci Biobehav Rev. 2014;47:445-56.
261. Sherrington C, Whitney JC, Lord SR, Herbert RD, et al. Effective Exercise for the Prevention of Falls: A Systematic Review and Meta-Analysis. J Am Geriatr Soc. 2008;56(12):2234-2243.
262. Siervo M, Arnold R, Wells JCK, Tagliabue A, et al. Intentional weight loss in overweight and obese individuals and cognitive function: a systematic review and meta-analysis. Obes Rev. 2011;12(11):968-983.
263. Sivan M, Sawyer C, Brown J. The role of exercise therapy in the secondary prevention of falls in elderly people. Int Musculoskelet Med. 2010;32(4):168-172.
264. Sjösten N, Vaapio S, Kivelä SL. The effects of fall prevention trials on depressive symptoms and fear of falling among the aged: A systematic review. Aging Ment Health. 2008;12(1):30–46.
265. Skelton DA, Howe TE, Ballinger C, Neil F, et al. Environmental and behavioural interventions for reducing physical activity limitation in community-dwelling visually impaired older people (Review). Cochrane Database Syst Rev. 2013;(6).
266. Smeeth LL, Iliffe S. Community screening for visual impairment in the elderly (Review). Cochrane Database Syst Rev. 2006;(3).
267. Smits CHM, de Lange J, Droes R-M, Meiland F, et al. Effects of combined intervention programmes for people with dementia living at home and their caregivers: a systematic review. Int J Geriatr Psychiatry. 2007; 22(12):1181-93.
268. Snowden M, Steinman L, Mochan K, Grodstein F, et al. Effect of Exercise on Cognitive Performance in Community-Dwelling Older Adults: Review of Intervention Trials and Recommendations for Public Health Practice and Research. J Am Geriatr Soc. 2011;59(4):704-716.
269. Song D, Shen Q, Xu TZ, Sun QH. Effects of group reminiscence on elderly depression: A meta-analysis. Int J Nurs Sci. 2014;1(4):416-422.
270. Stathokostas L, Little RMD, Vandervoort AA, Paterson DH. Flexibility Training and Functional Ability in Older Adults: A Systematic Review. J Aging Res. 2012;2012:306818.
271. Stehr MD, von Lengerke T. Preventing weight gain through exercise and physical activity in the elderly: A systematic review. Maturitas. 2012;72(1):13-22.
272. Stern C, Jayasekara R. Interventions to reduce the incidence of falls in older adult patients in acute-care hospitals: a systematic review. Int J Evid Based Healthc. 2009;7(4):243-249.
273. Stern C, Munn Z.. Cognitive leisure activities and their role in preventing dementia: a systematic review. Int J Evid Based Healthc. 2010;8(1):2-17.
274. Steultjens EMJ, Dekker J, Bouter LM, Jellema S, et al. Occupational therapy for community dwelling elderly people: a systematic review. Age Ageing. 2004;33(5):453-460.
275. Stevens Z, Barlow C, Kendrick D, Masud T, et al. Effectiveness of general practice-based physical activity promotion for older adults: systematic review. Prim Health Care Res Dev. 2014;15(2):190-201.
276. Stuck AE, Egger M, Hammer A, Minder CE, et al. Home Visits to Prevent Nursing Home Admission and Functional Decline in Elderly People. Systematic Review and Meta-regression analysis. JAMA. 2002;287(8):1022-1028.
277. Stuifbergen AK, Morris M, Jung JH, Pierini D, et al. Benefits of Wellness Interventions for Persons with Chronic and Disabling Conditions: A Review of the Evidence. Disabil Health J. 2010;3(3):133–145.
278. Sydenham E, Dangour AD, Lim WS. Omega 3 fatty acid for the prevention of cognitive decline and dementia (Review). Cochrane Database Syst Rev. 2012;(6).
279. Tanaka R, Ozawa J, Umehara T, Kito N, et al. Does Exercise Intervention Improve Muscle Strength and Balance of Japanese Subjects with Osteoporosis?: a Systematic Review and Meta-analysis of Randomized Controlled Trials. J Phys Ther Sci. 2013; 25(4): 397-401.
280. Tanaka R, Ozawa J, Umehara T, Kito N, et al. Exercise Intervention to Improve the Bone Mineral Density and Bone Metabolic Markers as Risk Factors for Fracture in Japanese Subjects with Osteoporosis: a Systematic Review and Meta-analysis of Randomized Controlled Trials. J Phys Ther Sci. 2012; 24(12): 1349-1353.
281. Tappenden P, Campbell F, Rawdin A, Wong R,et al. The clinical effectiveness and costeffectiveness of home-based, nurse-led health promotion for older people: a systematic review. Health Technol Assess. 2012;16(20):1-72.
282. Teixeira CVL, Gobbi LTB, Corazza DI, Stella F, et al. Non-pharmacological interventions on cognitive functions in older people with mild cognitive impairment (MCI). Arch Gerontol Geriatr. 2012;54(1):175-180.
283. Testad I, Corbett A, Aarsland D, Osland Lexow K, et al. The value of personalized psychosocial interventions to address behavioral and psychological symptoms in people with dementia living in care home settings: a systematic review. Int Psychogeriatr. 2014;26(7):1083-1098.
284. Theou O, Stathokostas L, Roland KP, Jakobi JM, et al. The Effectiveness of Exercise Interventions for the Management of Frailty: A Systematic Review. J Aging Res. 2011;2011:569194.
285. Thomas S, Mackintosh S, Halbert J. Does the ‘Otago exercise programme’ reduce mortality and falls in older adults?: a systematic review and meta-analysis. Age Ageing. 2010;39(6):681-687.
286. Thomas RE, Russell M, Lorenzetti D. Interventions to increase influenza vaccination rates of those 60 years and older in the community (Review). Cochrane Database Syst Rev. 2010;(9).
287. Thomas E, Smith JE, Forrester DA, Heider G, et al. The effectiveness of non-pharmacological multi-component interventions for the prevention of delirium in non-intensive care unit older adult hospitalized patients: a systematic review. JBI Database System Rev Implement Rep. 2014;12(4):180-232.
288. Thompson P, Lang L, Annells M. A systematic review of the effectiveness of in-home community nurse led interventions for the mental health of older persons. J Clin Nurs. 2008;17(11):1419-1427.
289. Tschopp M, Sattelmayer MK, Hilfiker R. Is power training or conventional resistance training better for function in elderly persons? A meta-analysis. Age Ageing. 2011;40(5):549-56.
290. Tseng CN, Gau BS, Lou MF. The Effectiveness of Exercise on Improving Cognitive Function in Older People: A Systematic Review. J Nurs Res. 2011;19(2):119-131.
291. Turner S, Arthur G, Lyons RA, Weightman AL, et al. Modification of the home environment for the reduction of injuries (Review). Cochrane Database Syst Rev. 2011;(2).
292. Udell JA, Zawi R, Bhatt DL, Keshtkar-Jahromi M, et al. Association Between Influenza Vaccination and Cardiovascular Outcomes in High-Risk Patients. JAMA. 2013;310(16):1711-1720.
293. Vagetti GC, Barbosa Filho VC, Moreira NB, de Oliveira V, et al. Association between physical activity and quality of life in the elderly: a systematic review, 2000-2012. Rev Bras Psiquiatr. 2014;36(1):76-88.
294. Valenzuela T. Efficacy of Progressive Resistance Training Interventions in Older Adults in Nursing Homes: A Systematic Review. J Am Med Dir Assoc. 2012;13(5):418-428.
295. van der Bij AK, Laurant MGH, Wensing M. Effectiveness of Physical Activity Interventions for Older Adults A Review. Am J Prev Med. 2002;22(2):120-133.
296. van Diest M, Lamoth CJC, Stegenga J, Verkerke GJ, et al. Exergaming for balance training of elderly: state of the art and future developments. J Neuroeng Rehabil. 2013;10:101.
297. van Haastregt J, Diederiks J, van Rossum E, de Witte L, et al. Effects of preventive home visits to elderly people living in the community: systematic review. BMJ. 2000;320(7237):754-758.
298. van Uffelen JGZ, Chin A, Paw MJM, Hopman-Rock M, van Mechelen W.. The Effects of Exercise on Cognition in Older Adults With and Without Cognitive Decline: A Systematic Review. Clin J Sport Med. 2008;18(6):486-500.
299. Van’t Leven, Prick A-EJC, Groenewoud JG, Roelofs PDDM, et al. Dyadic interventions for community-dwelling people with dementia and their family caregivers: a systematic review. Int Psychogeriatr. 2013;(25)10:1581–1603.
300. Vasse E, Vernooij-Dassen M, Spijker A, Rikkert MO, et al. A systematic review of communication strategies for people with dementia in residential and nursing homes. Int Psychogeriatr. 2010;22(2):189-200.
301. Verhagen AP, Immink M, van der Meulen A, Bierma-Zeinstra SMA. The efficacy of Tai Chi Chuan in older adults: a systematic review. Fam Pract. 2004;21(1):107-113.
302. Vlaeyen E, Coussement J, Leysens G, Van der Elst E, et al. Kim Delbaere. Characteristics and Effectiveness of Fall Prevention Programs in Nursing Homes: A Systematic Review and Meta-Analysis of Randomized Controlled Trials. J Am Geriatr Soc. 2015;63(2):211-221.
303. Vu T, Farish S, Jenkins M, Kelly H. A meta-analysis of effectiveness of influenza vaccine in persons aged 65 years and over living in the community. Vaccine. 2002;20(13-14):1831-1836.
304. Wang Ch, Collet JP, Lau JL.. The Effect of Tai Chi on Health Outcomes in Patients With Chronic Conditions. Arch Intern Med. 2004;164(5):493-501.
305. Warkentin LM, Das D, Majumdar SR, Johnson JA, et al. The effect of weight loss on health-related quality of life: systematic review and meta-analysis of randomized trials. Obes Rev. 2014;15(3):169-182.
306. Watkins I, Xie B. eHealth literacy interventions for older adults: a systematic review of the literature. J Med Internet Res. 2014;16(11):e225.
307. Wayne PM, Walsh JN, Taylor-Piliae RE, Wells RE, et al. Effect of Tai Chi on Cognitive Performance in Older Adults: Systematic Review and Meta-Analysis. J Am Geriatr Soc. 2014;62(1):25-39.
308. Weatherall M,. Multifactorial risk assessment and management programmes effectively prevent falls in the elderly. Evid Base Healthc Publ Health. 2004; 8(5):270-272.
309. Weatherall M. Prevention of falls and fall-related fractures in community-dwelling older adults: a meta-analysis of estimates of effectiveness based on recent guidelines. Intern Med J. 2004;34(3):102-108.
310. Weening-Dijksterhuis E, de Greef MHG, Scherder EJA, Slaets JPJ, et al. Frail institutionalized older persons: A comprehensive review on physical exercise, physical fitness, activities of daily living, and quality-of-life.. Am J Phys Med Rehabil. 2011;90(2):156-168.
311. Whear R, Abbott R, Thompson-Coon J, Bethel A, et al. Effectiveness of Mealtime Interventions on Behavior Symptoms of People With Dementia Living in Care Homes: A Systematic Review. J Am Med Dir Assoc. 2014;15(3):185-193.
312. Whelton SP, Chin A, Xin X, He J. Effect of Aerobic Exercise on Blood Pressure: A Meta-Analysis of Randomized, Controlled Trials. Ann Intern Med. 2002;136(7):493-503.
313. Wilkins S, Jung B, Wishart L, Edwards M, et al. The effectiveness of community-based occupational therapy education and functional training programs for older adults: A critical review. Can J Occup Ther. 2003;70(4):214-225.
314. Willis BC, Ndiaye SM, Hopkins DP, Shefer A. Improving Influenza, Pneumococcal Polysaccharide, and Hepatitis B Vaccination Coverage Among Adults <65 Years at High Risk. MMWR Morb Mortal Wkly Rep. 2005;54(5):1-11.
315. Wilson J, Micucci S. Interventions to Prevent the Recurrence of Elder Abuse. Effective Public Health Practice Project2003.
316. Windle G, Hughes D, Linck P, Russel I, et al. Is exercise effective in promoting mental well-being in older age? A systematic review. Aging Ment Health. 2010;14(6):652-669.
317. Windle G, Hughes D, Linck P, Morgan R, et al. Public health interventions to promote mental well-being in people aged 65 and over: systematic review of effectiveness and cost-effectiveness. Mental Well-being and Older People: Review of Effectiveness & Cost Effectiveness2007.
318. Winter H, Watt K, Peel NM. Falls prevention interventions for community-dwelling older persons with cognitive impairment: a systematic review. Int Psychogeriatr. 2013;25(2):215-227.
319. Winterton R, Warburton J. Models of care for socially isolated older rural carers: barriers and implications. Rural Remote Health. 2011;11(3):1678.
320. Witham MD, Avenell A. Interventions to achieve long-term weight loss in obese older people. A systematic review and meta-analysis. Age Ageing. 2010;39(2):176-184.
321. Woods B, Aguirre E, Spector AE, Orrell M. Cognitive stimulation to improve cognitive functioning in people with dementia (Review). Cochrane Database Syst Rev. 2012;(2).
322. Wutzler P, Hardt R, Knuf M, Wahle K. Targeted Vaccine Selection in Influenza Vaccination. Dtsch Arztebl Int. 2013;110(47):793–798.
323. Xu LJ, Meng Q, He SW, Yin XL, et al. The effects of health education on patients with hypertension in China: A meta-analysis. Health Educ J. 2014;73(2) 137–149.
324. Yamaoka K, Tango T. Efficacy of Lifestyle Education to Prevent Type 2 Diabetes. Diabetes Care. 2005;28(11):2780-2786.
325. Yang PY, Ho KH, Chen HC, Chien MY. Exercise training improves sleep quality in middle-aged and older adults with sleep problems: a systematic review. J Physiother. 2012;58(3):157-163.
326. You EC, Dunt D, Doyle C, Hsueh A. Effects of case management in community aged care on client and carer outcomes: a systematic review of randomized trials and comparative observational studies. BMC Health Serv Res. 2012;12:395.
327. Young J, Angevaren M, Rusted J, Tabet N. Aerobic exercise to improve cognitive function in older people without known cognitive impairment (Review). Cochrane Database Syst Rev. 2015;(4).
328. Young K, Bunn F, Trivedi D, Dickinson A. Nutritional education for community dwelling older people: A systematic review of randomised controlled trials. Int J Nurs Stud. 2011;48(6):751-780.
329. Zanotto T, Bergamin M, Roman F, Sieverdes JC, et al. Effect of Exercise on Dual-task and Balance on Elderly in Multiple Disease Conditions. Curr Aging Sci. 2014;7(2):115-136.
330. Zbikowski SM, Magnusson B, Pockey JR, Tindle HA, et al. A review of smoking cessation interventions for smokers aged 50 and older. Maturitas. 2012;71(2):131-141.
331. Zehnacker C, Bemis-Dougherty A.. Effect of Weighted Exercises on Bone Mineral Density in Post Menopausal Women A Systematic Review. J Geriatr Phys Ther. 2007;30(2):79-88.
332. Zheng G, Huang M, Liu F, Li S, et al. Tai Chi Chuan for the Primary Prevention of Stroke in Middle-Aged and Elderly Adults: A Systematic Review. Evid Based Complement Alternat Med. 2015;2015:1-18.
333. Zhu H, An R. Impact of home-delivered meal programs on diet and nutrition among older adults: A review. Nutr Health. 2013;22(2):89–103.
334. Zijlstra RGA, van Haastregt JCM, van Rossum E, van Eijk JTM, et al. Interventions to Reduce Fear of Falling in Community-Living Older People: A Systematic Review. J Am Geriatr Soc. 2007;55(4):603-615.
